# Prevalence of traditional Chinese medicine body constitutions in a large community-based study in Hangzhou, China

**DOI:** 10.1186/s13020-025-01268-x

**Published:** 2025-11-28

**Authors:** Yi-Hsuan Wu, Ji Wang, Ming-Hua Bai, Qi Wang, April Myers, Peng Gao, Elizabeth Delzell, Dan Huang, Fei Yang, Wei He, Shankuan Zhu, Ann W. Hsing

**Affiliations:** 1 Stanford Prevention Research Center, Department of Medicine, Stanford School of Medicine, Stanford University, Stanford, CA USA; 2 National Institute of Traditional Chinese Medicine Constitution and Preventive Medicine, Beijing University of Chinese Medicine, Beijing, China; 3 Binjiang Institute of Zhejiang University, Hangzhou, Zhejiang China; 4 Department of Nutrition and Food Hygiene, The Children’s Hospital, Zhejiang University School of Medicine, National Clinical Research Center for Child Health, Hangzhou, Zhejiang China; 5 Chronic Disease Research Institute, School of Public Health, School of Medicine, Zhejiang University, Hangzhou, Zhejiang China; 6 Department of Epidemiology and Population Health, Stanford School of Medicine, Stanford University, Stanford, CA USA; 7 Stanford Cancer Institute, Stanford School of Medicine, Stanford University, Stanford, CA USA

**Keywords:** Traditional Chinese medicine (TCM), Body constitution, Prevalence, Composite constitutions, Tendency

## Abstract

**Background:**

Traditional Chinese medicine (TCM) views body constitution as a foundational determinant of health and disease risk. Understanding the distribution of body constitutions across the population can help in developing personalized strategies to prevent disease, but few studies have examined composite constitutions or unbalanced tendencies.

**Methods:**

This cross-sectional study assessed the prevalence of singular and composite (multiple) TCM body constitutions and tendencies in a sample of adult residents of Hangzhou, China. We used the 2016 version of the Constitution in Chinese Medicine Questionnaire (CCMQ, 54 items) to classify participants into nine body constitutions and tendencies toward those body constitutions and examined variations in those distributions by demographics and selected lifestyle factors.

**Results:**

Among 8,665 participants aged 18–80 years, 74.2% had one or more body constitutions, and 25.8% had unbalanced tendencies only. The Balanced constitution was the most common (22.3%). Of the eight unbalanced constitutions, Qi Deficiency (16.4%), Phlegm Dampness (11.0%), and Yang Deficiency (9.4%) were most prevalent. Over half of the individuals with an unbalanced constitution also had other constitutions (composite). The most frequent composite combinations included Qi Stagnation with Qi Deficiency, Blood Stasis and Qi Deficiency, and Damp Heat with Phlegm Dampness. Body constitution distribution varied significantly by age, sex, and body mass index (BMI). Younger adults (18–39 years) were less likely to have the Balanced constitution (13.1%) and more likely to have composite unbalanced constitutions. Men were more likely to have Phlegm Dampness or Damp Heat, while women were more likely to have Yang Deficiency or Yin Deficiency. After adjusting for age and sex, individuals with obesity had a higher prevalence of Phlegm Dampness and a lower prevalence of the Balanced, Yang Deficiency, or Yin Deficiency constitutions.

**Conclusions:**

Our results provide a comprehensive profile of the patterns and distribution of TCM body constitutions across demographic and lifestyle subgroups. This more complete understanding of TCM body constitutions can inform personalized medicine, support individual risk assessment, and help improve health outcomes.

**Supplementary Information:**

The online version contains supplementary material available at 10.1186/s13020-025-01268-x.

## Introduction

In traditional Chinese medicine (TCM), body constitution refers to a person’s natural health foundation, primarily inherited at birth, but shaped over time by lifestyle, diet, emotions, and environment [1]. The concept of body constitution originated more than two thousand years ago in Huangdi Neijing’s *Yellow Emperor’s Canon of Medicine* [2] and remains central to TCM theory and practices. Body constitution influences how individuals respond to internal and external factors such as illness, stress, and seasonal changes. When the body constitution is balanced, individuals are thought to function optimally and maintain resistance to disease. However, when the balance is disrupted, by factors such as stress, poor sleep, or unhealthy dietary patterns, individuals may become more susceptible to diseases [1]. Different types of imbalances are associated with distinct physiological, anatomical, and psychological characteristics and disease susceptibility. To establish a framework for this long-standing concept, the China Association of Chinese Medicine published “Classification and Determination of Constitution in TCM” in 2009 [3]. These guidelines introduced a clinically validated algorithm that classifies individuals into nine body constitutions based on their inherent traits. These include the Balanced constitution, representing a state of energetic harmony and physical and mental well-being, and eight unbalanced constitutions: Qi Deficiency, Yin Deficiency, Yang Deficiency, Phlegm Dampness, Damp Heat, Blood Stasis, Qi Stagnation, and Inherited Special constitutions.

One’s TCM body constitution influences overall health, susceptibility to certain diseases, and response to medical treatments [4, 5]. Certain unbalanced body constitutions have been found to be associated with a wide range of chronic diseases, including metabolic and cardiovascular disorders, various cancers, respiratory illness, gastrointestinal diseases, reproductive disorders, and mental health conditions [6]. Body constitutions are affected by both innate (genetic) and acquired (environmental, lifestyle) factors [1], but they tend to remain relatively stable, making them useful for predicting disease risk and tailoring health practices. However, one can modify one’s body constitution and improve health outcomes by making sustained positive environmental or lifestyle changes [2].

Having a better understanding of the patterns and prevalences of the TCM body constitutions in a population could help in developing effective and personalized strategies to prevent disease and improve health outcomes in individuals and communities. Although there have been some population-based studies in this area, most have been confined to particular demographics, such as male or female [7, 8], elderly individuals [9, 10], or certain patient populations [11, 12]. Two large-scale studies have provided valuable insights into TCM body constitution distribution in the general Chinese population, both using the 2006 version of the Constitution in Chinese Medicine Questionnaire (CCMQ, 60 items). The first, conducted between 2005 and 2007 in nine provinces and municipalities across China, surveyed 8,448 individuals and found one-third had the Balanced constitution (32.1%), with Qi Deficiency (13.4%), Damp Heat (9.1%), and Yang Deficiency (9.0%) as the most common unbalanced constitutions [13]. The second, a nationwide internet-based survey conducted from 2015 to 2017 with 108,015 participants, reported a Balanced constitution prevalence of 28.9%, with Yang Deficiency (16.4%), Qi Deficiency (13.2%), and Damp Heat (10.2%) as the most common unbalanced constitutions, differing by sex, age, and region [14].

These two large-scale studies examined only a single primary constitution for each individual. However, previous studies have suggested that 50–60% of individuals may have multiple coexisting body constitutions, or “composite constitutions” [15, 16]. Having multiple unbalanced constitutions may indicate greater physiological and psychological imbalance, leading to increased susceptibility to various physical and mental health conditions [17]. Additionally, the 2009 “Classification and Determination of Constitution in TCM” guidelines suggest that individuals may have tendencies toward specific unbalanced constitutions, even if they cannot be fully classified into them [3]. Approximately 20% of individuals have these unbalanced tendencies [17], which may signal emerging imbalances and provide opportunities for early intervention. Although composite constitutions and tendencies are common and clinically relevant, no prior studies have examined composite constitution patterns or reported the prevalence of unbalanced tendencies in a large population-based setting.

To address this gap, we used data from the Wellness Living Laboratory China (WELL China) cohort, a large community-based study established through a collaboration between Stanford University, in the United States, and Zhejiang University, in Hangzhou, Zhejiang Province, China [18], to assess the prevalence of each of the nine TCM body constitutions and tendencies. This analysis examined how these distributions varied across demographic and lifestyle groups and investigated patterns of composite constitutions and tendencies.

## Methods

### Study population

We conducted a cross-sectional analysis of baseline data from the WELL China study. WELL China enrolled 10,268 participants aged 18–80 years from three districts (Xihu, Shangcheng, and Gongshu) in Hangzhou between 2016 and 2019. To ensure a representative population distribution, long-term residents were identified and sampled using quota sampling based on age and sex for each subdistrict and community [18]. All participants provided informed consent. The WELL China study was approved by the Institutional Review Boards at both Stanford University (IRB-35020) and Zhejiang University (ZGL201507-3).

### Data collection

Details of data collection have been described previously [18]. Briefly, we conducted an in-person survey with approximately 1,000 questions, covering the following self-reported data components: demographic characteristics, lifestyle behaviors, medical history, well-being (Stanford WELL for Life Scale) [19], and TCM body constitution. TCM body constitution was assessed using the 2016 version of the Constitution in Chinese Medicine Questionnaire (CCMQ) [20], a validated instrument designed to classify individuals into one or more of nine body constitutions based on physical, psychological, and behavioral traits [21, 22]. The 2016 version of CCMQ contains 54 items that are administered as 57 questions (Table S1), because the allergic-related item is assessed using four separate questions. In addition, we collected anthropometric measurements and whole blood samples for laboratory tests of biochemical markers as indicators of chronic conditions.

### Classification of TCM body constitution

To classify each individual’s body constitution, we applied the “Classification and Determination of Constitution in TCM” (ZZYXH/T157-2009), a standard and clinically validated algorithm released by the China Association of Chinese Medicine [3]. Each question was related to a specific body constitution: Balanced constitution (7 questions), Qi Deficiency (6), Yin Deficiency (6), Yang Deficiency (7), Phlegm Dampness (5), Damp Heat (6), Blood Stasis (7), Qi Stagnation (7), and Inherited Special (6). For each body constitution, we summed the response numbers for the related questions to obtain a raw score. This raw score was then converted using the following formula, where N represents the number of questions [3]:

$$Converted Score = \left( {\frac{Raw Score - N}{{N \times 4}}} \right) \times 100$$

Each individual had nine converted scores, one for each constitution, ranging from 0 to 100. The converted scores were then used to classify individuals according to the following body constitution/tendency categories [3]:

- Balanced constitution: If the Balanced score was 60 or higher and all eight other constitution scores were below 30, the individual was classified as having a Balanced constitution. If the Balanced score was below 60 or any of the eight other constitution scores was 30 or higher, the individual was not classified as having a Balanced constitution.
- Unbalanced constitution: If the score for an unbalanced constitution was 40 or higher, the individual was classified as having that unbalanced constitution.
- Unbalanced tendency: If the score for an unbalanced constitution was in the 30–39 range, the individual was classified as having a tendency toward that constitution.
- No classification: If the Balanced score was below 60 and all unbalanced constitution scores were below 30, the individual was not classified as having any body constitution or tendency.

After each individual was assigned one or more body constitution category, they were classified into one of the following three groups:

1. Body constitution(s): Individuals with at least one body constitution. This group was further divided into:
2. Balanced constitution: Individuals with a Balanced constitution only.
3. Unbalanced constitution: Individuals with at least one unbalanced constitution. They may also have unbalanced tendencies. For those with a single unbalanced constitution, their primary classification was that constitution. For those with multiple (composite) constitutions, the constitution with the highest score was considered their primary classification.
4. Only unbalanced tendency(s): Individuals who did not have any body constitution but had at least one unbalanced tendency. For those with a single tendency, this was their primary classification. For those with multiple tendencies, the tendency with the highest score was considered their primary classification.
5. No classification**:** Individuals who did not have any body constitution or tendency.

The full classification process is illustrated in Figure S1, which provides a visual summary of the decision rules described above and an example of its application.

### Exclusions

Figure S2 includes a flow chart of participants included in further analyses. Among the 10,268 participants enrolled in the WELL China study, we excluded 1,119 (10.9%) participants who did not respond to the 2016 version of the CCMQ and nine (0.1%) participants who had missing responses to any of the 57 TCM questions, as the body constitution classification algorithm requires complete responses to calculate body constitution converted scores. This resulted in 9,140 participants with complete responses to all TCM questionnaire items.

The CCMQ uses five-point Likert-type questions, with “sometimes” as the middle response (Table S1). While selecting the middle response can reflect genuine neutrality, choosing this response for many questions (> 50%) may reflect low engagement or difficulty fully understanding the questions, potentially resulting in a score with low reliability and a high chance of misclassification [23, 24]. To address this, we examined the distribution of middle responses across the 9,140 participants. We excluded 16 (0.2%) participants who selected the middle response for 50 or more questions (88% of the 57 questions) (Figure S3). Of the 16, 13 had all eight unbalanced constitutions, a pattern that is biologically implausible and may reflect misclassification. To assess the robustness of the results after removing these participants, we conducted sensitivity analyses (1) without excluding participants based on middle responses, and (2) excluding those who selected the middle response for 29 (≥ 50%) or more questions. Results were consistent across both analyses (Figure S4), suggesting that our findings are robust to varying levels of stringency in the exclusion criteria.

Of the remaining 9,124 participants with complete TCM data, 459 (5.0%) were excluded because their total scores did not meet the criteria for a specific body constitution classification. Their Balanced constitution scores were below 60, and all eight unbalanced scores were below 30. This left 8,665 participants (84.4%) in the final analysis. The 459 excluded participants had lower education levels, lower income, and were more likely to be working (Table S2).

Of the 1,119 (10.9%) excluded participants who did not respond to the 2016 version of the CCMQ, 763 (68.2%) had completed an earlier version of the questionnaire. Compared to the analytic sample (N = 8,665), those who took an earlier version of the questionnaire (n = 763) had a lower prevalence of the Balanced constitution (15.6% vs. 22.3%) (Figure S5). This observation may reflect differences in the structure of the two questionnaires or scoring algorithms. Thus, in this analysis, we limited the samples to the 8,665 participants who completed the 2016 version of the CCMQ.

### Statistical analysis

We used WELL China participants’ baseline data for demographics (sex, age, education, income, work status, and marital status), smoking history, alcohol consumption, and body mass index (BMI). For smoking status, participants were categorized as never, former, or current smokers. Alcohol consumption was categorized as never, former, current occasional, and current frequent drinkers (12 times or more per year). We divided participants into four BMI groups: underweight (< 18.5 kg/m^2^), normal (18.5–23.9 kg/m^2^), overweight (24–27.9 kg/m^2^), and obese (≥ 28 kg/m^2^), based on the cutoffs recommended by the Working Group on Obesity in China [25].

### Estimation of primary constitution prevalence

We calculated the prevalence of each body constitution and tendency as the number of participants with that specific classification divided by the total number of participants in our analytic sample (N = 8,665). Prevalence was calculated for both overall and within each demographic and lifestyle category. To assess whether the prevalence of body constitutions differed by age or sex, we used chi-squared tests. We also used chi-squared tests to evaluate whether there were linear trends in the prevalence of specific body constitutions across age groups. Statistical significance was determined at a Bonferroni-corrected *p* value of 0.05/9 = 0.006.

To evaluate the associations between demographic and lifestyle factors and body constitution, we used modified Poisson regression with robust standard errors, adjusting for potential confounders (age and sex) [26]. This approach estimates the adjusted prevalence ratio (PR) and its 95% confidence interval (CI). We also used this model to estimate the adjusted prevalence for each subgroup using marginal standardization (also known as estimated marginal means) [27].

### Calculation of proportion of composite constitutions

To understand how different unbalanced constitutions coexist, we calculated, for each primary constitution, the proportion of participants who also had another specific unbalanced constitution. For example, among participants with Qi Stagnation, we calculated the percentage who also had Qi Deficiency. Since there are many possible combinations, we used a chord diagram to visually summarize these relationships in a clear and interpretable way.

All data analyses were conducted using R version 4.3.2 and RStudio version 2023.09.1 + 494 (Posit Software, PBC, Boston, MA, USA).

## Results

### Demographic and lifestyle characteristics

The demographic and lifestyle characteristics of the 8,665 eligible participants are shown in Table 1. Approximately 80% of the participants were between the ages of 30 and 70, and 61% were female. Those who had completed only middle school constituted the largest education group (31.7%). About 70% reported an annual income of $3,000–$12,000 USD, and half were retired. Most participants were married (87.1%), 74.5% were never smokers, and 54.0% were never drinkers. The distribution of these characteristics was similar between the 6,431 (74.2%) participants with a specific body constitution and the 2,234 (25.8%) participants with only unbalanced tendencies.

**Table 1:** Demographic and lifestyle characteristics of WELL China participants with TCM classifications (N = 8,665)

Characteristics	Total	Have body constitution(s)	Have only unbalanced tendency(s)
	n (%)	n (%)	n (%)
All	8665 (100.0)	6431 (74.2)	2234 (25.8)
Age			
18–29	467 (5.4)	372 (5.8)	95 (4.3)
30–49	2374 (27.4)	1814 (28.2)	560 (25.1)
50–59	2233 (25.8)	1616 (25.1)	617 (27.6)
60–69	2588 (29.9)	1885 (29.3)	703 (31.5)
70 +	1003 (11.6)	744 (11.6)	259 (11.6)
Sex			
Male	3383 (39.0)	2486 (38.7)	897 (40.2)
Female	5282 (61.0)	3945 (61.3)	1337 (59.8)
Education			
Elementary school and under	1669 (19.3)	1228 (19.1)	441 (19.7)
Middle school	2751 (31.7)	1980 (30.8)	771 (34.5)
High school	2062 (23.8)	1507 (23.4)	555 (24.8)
College and above	2183 (25.2)	1716 (26.7)	467 (20.9)
Annual income (USD)			
< $3104	1034 (11.9)	792 (12.3)	242 (10.8)
$3104–$7760	4417 (51.0)	3218 (50.0)	1199 (53.7)
$7760–$12,416	1978 (22.8)	1481 (23.0)	497 (22.3)
$12,416 +	1234 (14.2)	939 (14.6)	295 (13.2)
Work status			
Working	2533 (29.2)	1904 (29.6)	629 (28.2)
Others	1862 (21.5)	1413 (22.0)	449 (20.1)
Retired	4270 (49.3)	3114 (48.4)	1156 (51.7)
Marital status			
Married	7551 (87.1)	5573 (86.7)	1978 (88.5)
Others	633 (7.3)	476 (7.4)	157 (7.0)
Single	481 (5.6)	382 (5.9)	99 (4.4)
Smoking status			
Never	6456 (74.5)	4807 (74.8)	1649 (73.8)
Former	620 (7.2)	448 (7.0)	172 (7.7)
Current	1588 (18.3)	1175 (18.3)	413 (18.5)
Alcohol drinking status			
Never	4677 (54.0)	3485 (54.2)	1192 (53.4)
Former	169 (2.0)	121 (1.9)	48 (2.1)
Current occasionally	2160 (24.9)	1620 (25.2)	540 (24.2)
Current frequent	1659 (19.1)	1205 (18.7)	454 (20.3)
Body mass index (BMI)			
< 18.5 (Underweight)	328 (3.8)	267 (4.2)	61 (2.7)
18.5–23.9 (Normal)	4503 (52.0)	3331 (51.9)	1172 (52.5)
24.0–27.9 (Overweight)	2995 (34.6)	2185 (34.0)	810 (36.3)
28 + (Obese)	826 (9.5)	637 (9.9)	189 (8.5)

### Prevalences of primary body constitutions and tendencies

**Fig. 1:**
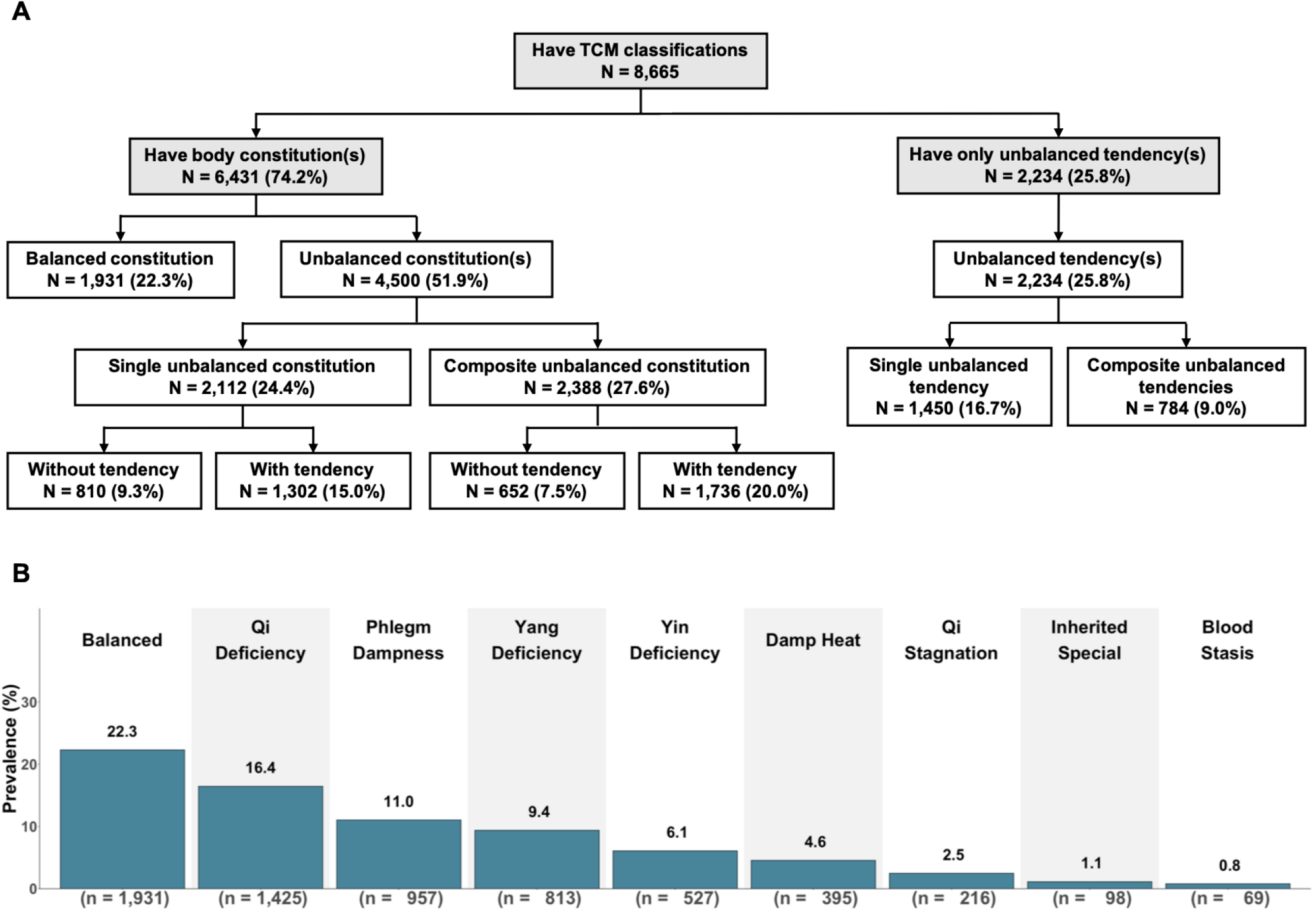
TCM body constitutions and tendencies among WELL China participants with TCM classifications (N = 8,665). **A** Distribution of participants based on presence of body constitution(s) and/or unbalanced tendency(ies). **B** Prevalence of each primary body constitution among 8,665 participants

Among the 8,665 participants, 22.3% had the balanced constitution, while over three-quarters of the participants had either unbalanced constitutions (51.9%) or unbalanced tendencies (25.8%) (Fig. 1A). Those with unbalanced constitutions included two distinct subgroups, those with a single unbalanced constitution (24.4% of total participants) and those with composite unbalanced constitutions (27.6% of all participants). Notably, only 9.3% of participants had a single unbalanced constitution without any accompanying tendency.

The prevalences of the primary body constitutions among the 8,665 participants are shown in Fig. 1B. Overall, Qi Deficiency was the most common unbalanced constitution (16.4%), followed by Phlegm Dampness (11.0%), Yang Deficiency (9.4%), Yin Deficiency (6.1%), Damp Heat (4.6%), Qi Stagnation (2.5%), Inherited Special (1.1%), and Blood Stasis (0.8%). The ranking of unbalanced tendencies followed a similar pattern, with Qi Deficiency being the most prevalent (9.6%) (Figure S6).

### Composite body constitutions and coexistence patterns

**Fig. 2:**
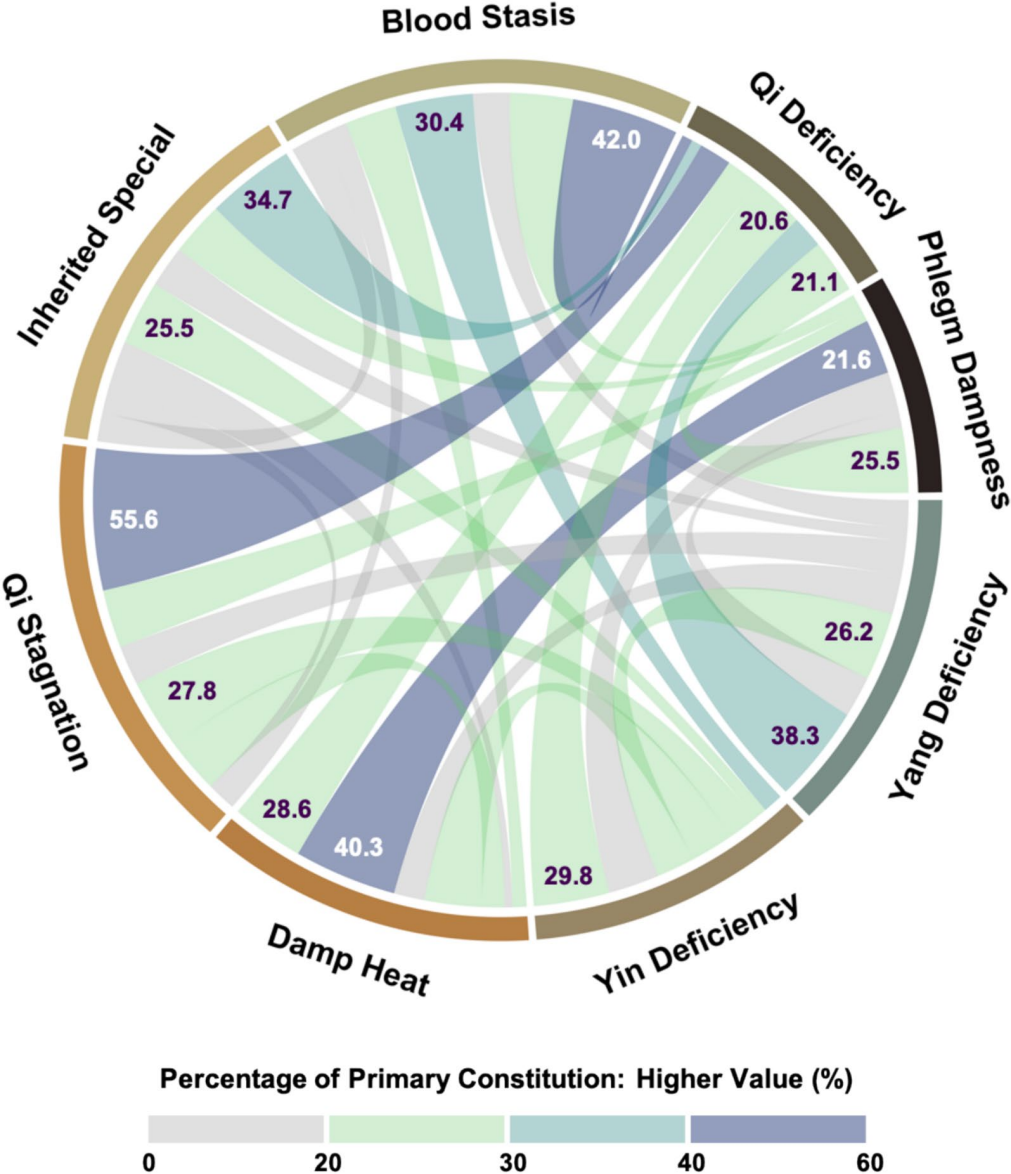
Percentage of coexisting body constitutions by primary constitution among participants with unbalanced constitutions (N = 4,500). Each segment around the circle represents one of the eight unbalanced constitutions. Chords connecting two segments indicates the coexistence of two constitutions. The width of each chord represents the percentage of participants with a coexisting body constitution, calculated within participants with the specified primary constitution. The width of the two ends of a chord may differ, as the percentage of participants with constitution A who also have constitution B is not necessarily equal to the percentage of participants with constitution B who also have constitution A. Chord color is based on the higher of the two percentages, with darker shades indicating higher values.

Over half of the participants with a primary unbalanced constitution (53.1%) also had other constitutions (composite) (Table 2). Individuals with the least common primary constitutions were more likely to have composite constitutions. For example, only 2.5% of the participants had a primary Qi Stagnation, but 72.2% of these individuals also had another unbalanced constitution. Specifically, of the 216 individuals with Qi Stagnation, 55.6% also had Qi Deficiency, and 27.8% had Yin Deficiency (Fig. 2). Similarly, for the two other uncommon constitutions, Inherited Special (1.1%) and Blood Stasis (0.8%), over 60% of them also had composite constitutions, most often coexisting with Qi Deficiency and Yin Deficiency. For the remaining five unbalanced constitutions, 40–60% of individuals had composite constitutions. Notably, Damp Heat frequently coexisted with Phlegm Dampness (40.3%), while Yang Deficiency often coexisted with Qi Deficiency (38.3%).

**Table 2:** Proportion with composite constitutions or unbalanced tendencies by primary body constitution among participants with unbalanced constitutions (N = 4,500)

Primary constitution	Prevalence	With composite constitutions	With unbalanced tendencies
	n (%)	n (%)	n (%)
All unbalanced constitutions	4500 (51.9)	2388 (53.1)	3038 (67.5)
Qi deficiency	1425 (16.4)	692 (48.6)	927 (65.1)
Phlegm dampness	957 (11.0)	417 (43.6)	595 (62.2)
Yang deficiency	813 (9.4)	505 (62.1)	562 (69.1)
Yin deficiency	527 (6.1)	278 (52.8)	381 (72.3)
Damp heat	395 (4.6)	231 (58.5)	292 (73.9)
Qi stagnation	216 (2.5)	156 (72.2)	166 (76.9)
Inherited special	98 (1.1)	62 (63.3)	64 (65.3)
Blood stasis	69 (0.8)	47 (68.1)	51 (73.9)

The proportion of composite constitutions varied significantly by age, with younger participants (aged 18–39 years) more likely to have composite constitutions compared to older participants (p < 0.001) (Table S3). While coexistence patterns were generally consistent across age groups, younger adults exhibited a greater complexity, with nine constitution pairs reaching at least a 40% coexistence proportion compared to just one pair in older adults (Figure S7).

In addition to the high proportion of composite constitutions, 67.5% of those with unbalanced constitutions also had one or more unbalanced tendencies. This proportion that remained consistently high across all primary constitutions, ranging from 60 to 75% (Table 2).

### Prevalences of primary body constitutions by age and sex

**Fig. 3:**
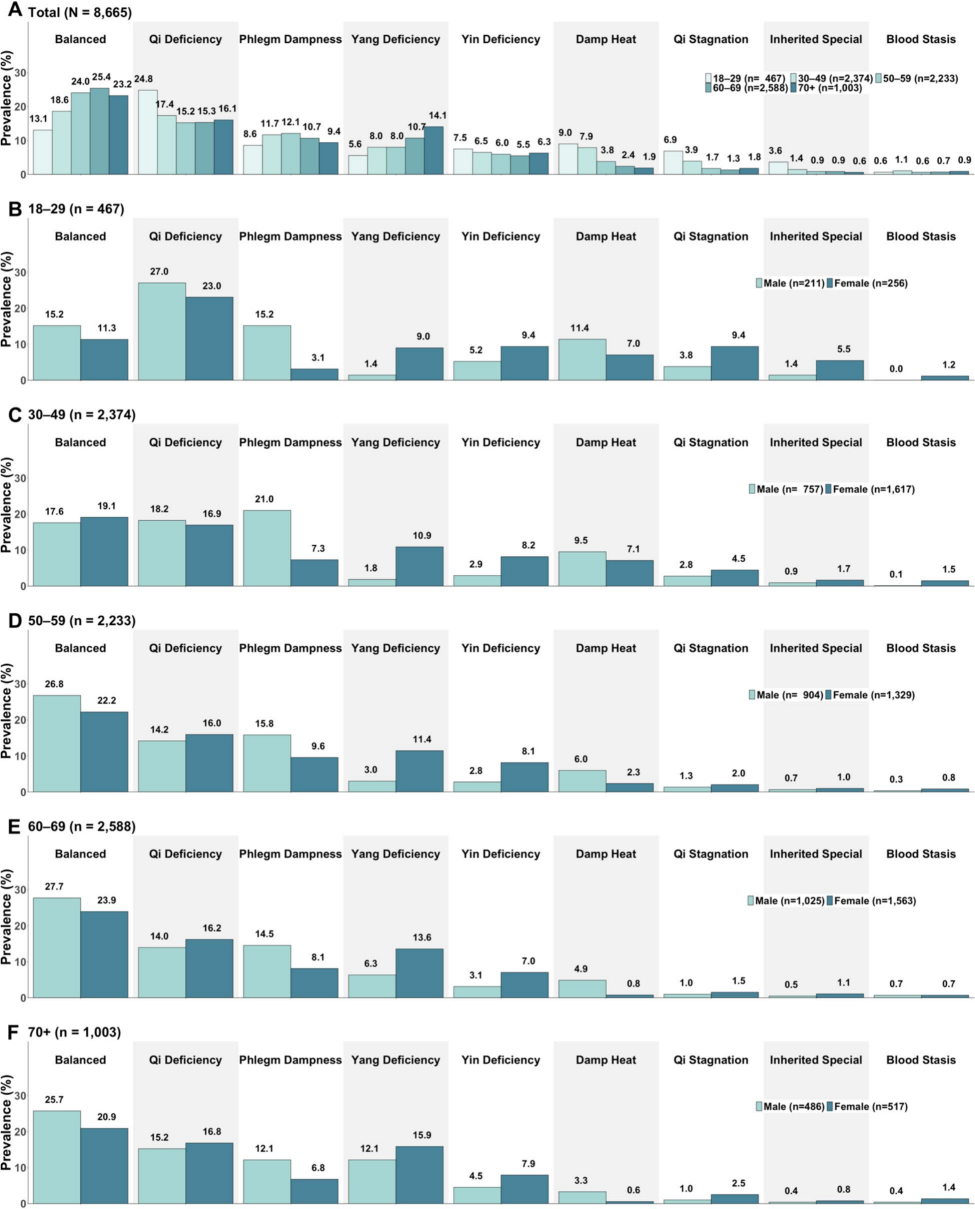
Prevalence of primary body constitutions by age and sex (N = 8,665). **A** Prevalence of primary body constitution by age. **B**–**F** Prevalence of primary body constitution by sex within each age group

Body constitution prevalence varied by age and sex. The prevalence of Balanced constitution was lowest among the youngest group (aged 18–29; 13.1%) and highest among those aged 60–69 (25.4%) (Fig. 3A). The youngest age group had the highest prevalences of Qi Deficiency (24.8%), Qi Stagnation (6.9%), and Inherited Special (3.6%) constitutions, all of which decreased significantly with age. Older participants were more likely to have Yang Deficiency, particularly those aged 70 years or older (14.1%). All the age differences mentioned here were statistically significant (*p* < 0.001).

For most age groups, males had a slightly higher prevalence of the Balanced constitution than females (24.1% vs. 21.1%, *p* = 0.001) (Fig. 3B–F). Phlegm Dampness (16% vs. 7.9%, *p* < 0.001) and Damp Heat (6.4% vs. 3.4%, *p* < 0.001) were more common in males across nearly all age groups. In contrast, Yang Deficiency (12.2% vs. 5.0%, *p* < 0.001) and Yin Deficiency (7.9% vs. 3.3%, *p* < 0.001) were more prevalent in females across most age groups.

### Prevalences of primary body constitutions by other factors

**Fig. 4:**
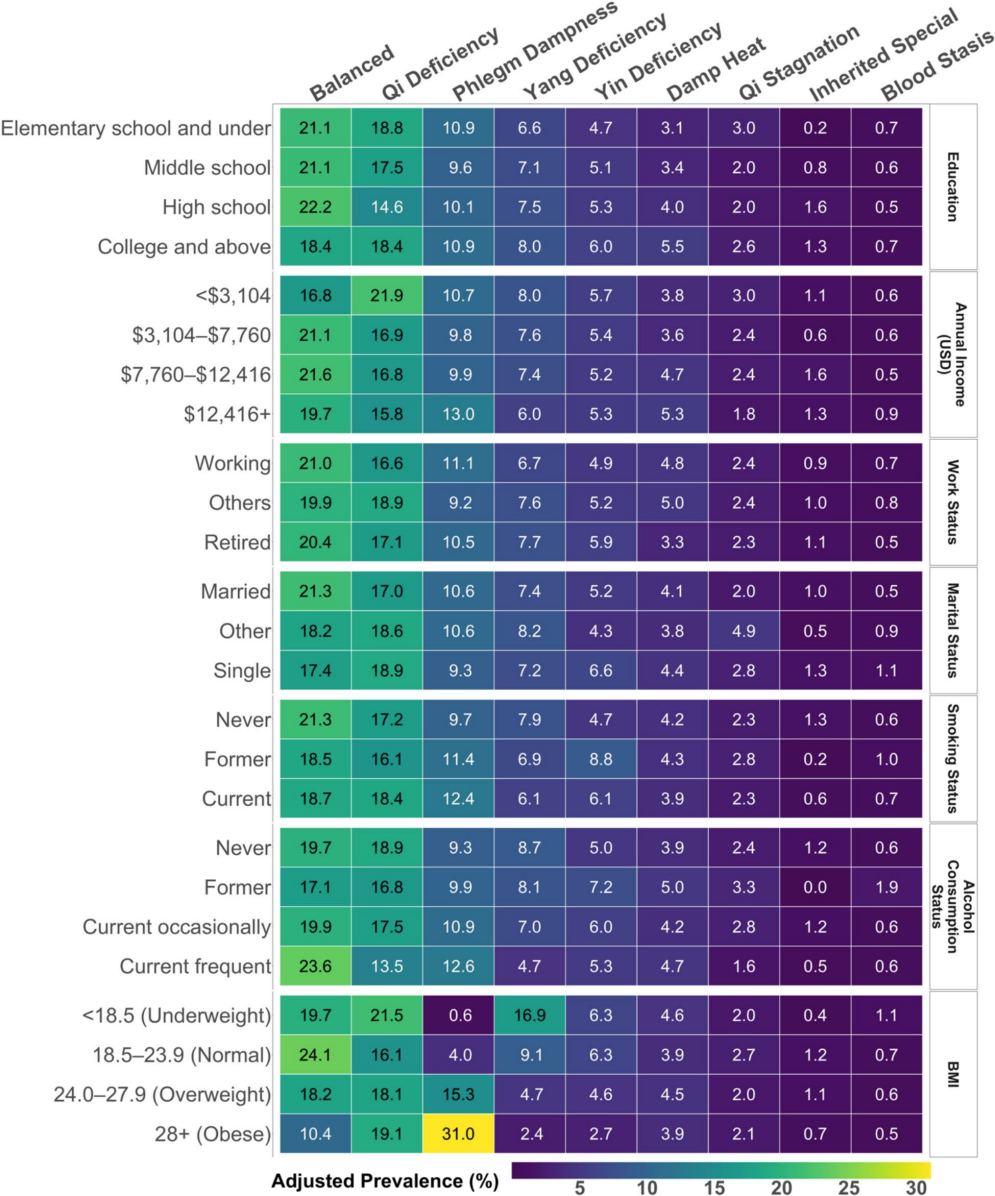
Adjusted prevalence of primary body constitutions by demographic and lifestyle characteristics (N = 8,665). The heatmap displays the adjusted prevalence (%) of each primary body constitution within each subgroup of demographic or lifestyle factors (e.g. married, other, or single within marital status). Adjusted prevalences were calculated as adjusted marginal means from modified Poisson regression models, with all models adjusted for age and sex.

Figure 4 presents the prevalences of primary body constitutions across demographic and lifestyle characteristics after adjusting for age and sex (details in Table S4). While most factors showed modest variations in constitution prevalence, differences by BMI were more pronounced. Individuals with obesity had the lowest prevalence of the Balanced constitution (10.4% vs. 24.1%). Phlegm Dampness increased sharply with BMI, peaking at 31.0% in individuals with obesity, while Yang Deficiency (2.4% vs. 16.9%) and Yin Deficiency (2.7% vs. 6.3%) decreased with higher BMI. All differences and trends described here were statistically significant (*p* < 0.001). These BMI-related patterns were observed across all age groups (Table S5) and in both males and females (Table S6).

## Discussion

In this large community-based study of 8,665 participants in Hangzhou, China, we found that the Balanced constitution was the most common body constitution (22.3%), while 77.7% of participants had one or more unbalanced constitutions or tendencies. Among those with unbalanced constitutions, more than half had composite constitutions. Younger adults (18–39 years) were less likely to have the Balanced constitution and more likely to have composite unbalanced constitutions. It has been suggested that individuals with different body constitution have different disease predisposition, and having composite unbalanced constitutions could lead to higher susceptibility to disease [17]. Thus, more accurate and complete information on TCM body constitutions can be integrated with genomics and lifestyle data to inform personalized medicine, to assess individual’s risk, and to improve and optimize their health outcomes.

Our findings were generally consistent with those from a large nationwide study conducted in China between 2015 and 2017 [14], which reported that the prevalence of the Balanced constitution increased with age, and males were more likely to have Phlegm Dampness and Damp Heat body constitutions, while females more commonly had Yang Deficiency. While these subgroup patterns aligned, overall, our study found higher prevalences of Qi Deficiency (16.4% vs. 13.2%) and Phlegm Dampness (11.0% vs. 6.8%), but lower prevalences of Balanced constitution (22.3% vs. 28.8%), Damp Heat (4.6% vs. 10.2%), Qi Stagnation (2.5% vs. 6.6%), Inherited Special (1.1% vs. 2.6%), and Blood Stasis (0.8% vs. 8.1%). These differences may be related to variations in survey version (we used the 2016 version) survey administration (in-person vs. online), geography (urban vs. national), lifestyle, and age distribution of study participants (mean age 54.3 in our study vs. 44.7 in the other).

It is noteworthy that three in four people had an unbalanced constitution, especially among young adults (age < 30 years, 87%), suggesting that more health and TCM education on healthy lifestyles and prevention strategies are needed in this segment of the population. Moreover, younger adults were 20% more likely to have composite unbalanced constitutions, further exacerbating their disease risk. The observation that young adults were more likely to have unbalanced constitutions, especially those that have been linked to chronic diseases such as Qi Deficiency and Qi Stagnation [6], is concerning given the rising incidence of obesity and early onset of cancer and other chronic disease in this population worldwide [28, 29]. These patterns highlight the potential utility of constitution-based assessment as a tool for identifying at-risk individuals and implementing preventive strategies that support long-term health and well-being in younger populations.

The large sample size of the study allowed us to comprehensively investigate composite body constitutions, an underexplored but clinically relevant dimension of TCM [30]. Among participants with unbalanced constitutions, over half (53.1%) had composite unbalanced constitutions, underscoring the importance of considering multiple coexisting unbalanced body constitutions rather than focusing on a primary constitution exclusively in research and clinical settings. We confirmed several patterns of constitution coexistence, such as the coexistence of Damp Heat with Phlegm Dampness, Qi Stagnation with Qi Deficiency [15] and Qi Deficiency with Yang Deficiency [31]. It is intriguing that composite unbalanced constitutions were most common among the three rarest primary body constitutions. For example, although Qi Stagnation consists of 2.5% of the primary body constitutions, most (72.2%) also had another constitution. The underlying biology or interactions of these concomitant constitutions is unclear, but it is important to consider composite body constitutions when treating individuals with these rare body constitutions to improve health and disease outcomes. Many of these pairings align with TCM theory. For example, Qi Stagnation and Qi Deficiency are thought to have a bidirectional relationship, where Qi Deficiency weakens Qi movement, and Qi Stagnation impairs Qi production [32]. Similarly, Qi Deficiency can slow blood flow and contributes to the development of Blood Stasis [33], a TCM condition often linked to cardiovascular disease [34]. These findings highlight the need for further research on how composite constitutions interact and influence health outcomes.

Key strengths of our study are its large, well-characterized sample and its use of a validated TCM survey to assess body constitutions and tendencies across diverse demographic and lifestyle factors. Our detailed analysis of the coexistence of various constitutions, particularly those less prevalent, such as Qi Stagnation, Inherited Special, and Blood Stasis, supports the need for investigation of the joint effects of multiple unbalanced constitutions in health outcomes in future studies. Limitations of the study should be noted. First, we excluded 11% of participants who did not complete the 2016 TCM questionnaire, 5% with no specific body constitution classification, 0.1% missing one or more TCM responses, and 0.2% with excessive middle responses. These exclusions potentially could result in selection bias, as excluded participants had lower education levels, lower income, and a higher prevalence of working, which were related to body constitutions. However, other than the ineligibles, selection bias in most exclusions is likely minimal as the sample size is quite small (< 1%). Second, the study was conducted in Hangzhou, an urban area of China, limiting generalizability to other populations. Lastly, this cross-sectional study does not capture longitudinal changes in body constitutions, although these are generally quite stable over time [1].

Understanding the distribution and complexity of TCM body constitutions, singular and composite, in the general population is an important first step toward integrating constitution-based insights into effective public health and personalized prevention approaches. Future studies can investigate how composite constitutions and tendencies’ relation to health outcomes and their potential value in predicting disease risk. The diversity of these patterns presents both challenges and opportunities for developing tailored preventive or therapeutic strategies. Continued investigations into how composite constitution patterns interact with health determinants and how these patterns evolve across the life course will be essential to unlocking the full potential of TCM body constitution theory in personalized medicine and population health.

## Conclusions

Our study presents a comprehensive evaluation of the prevalence and coexistence of TCM body constitutions in a large, community-based sample from Hangzhou, a major urban center in eastern China. We provide detailed information on body constitution distributions overall and across different demographic and lifestyle groups, highlighting key patterns of composite constitutions and unbalanced tendencies. These findings provide valuable insights to inform public health strategies for disease prevention based on TCM principles.

## Supplementary Information

Supplementary material 1.

## Data Availability

The data supporting the findings of this study are available from the corresponding author upon reasonable request.

## Abbreviations

TCM: Traditional Chinese medicine
BMI: Body mass index
PR: Prevalence ratio
