# Supplementary material for: Prevalence of traditional Chinese medicine body constitutions in a large community-based study in Hangzhou, China
Source: Chin Med. 2025 Nov 28;20:206. doi: 10.1186/s13020-025-01268-x (PMC12661724; doi:10.1186/s13020-025-01268-x)
Supplement: Supplementary file 1 — Supplementary material 1. [file 13020_2025_1268_MOESM1_ESM.docx]

**Supplementary Data**

**Supplementary Figure S1. Decision tree for classifying TCM body constitution and tendency.**

For example, a participant with a Balanced score of 79, a Qi Deficiency score of 67, a Yin Deficiency score of 38, and a Damp Heat score of 46 would be classified as having both Qi Deficiency and Damp Heat constitutions, with a Yin Deficiency tendency. Qi Deficiency would be considered the primary constitution, as it had the highest score among the unbalanced constitutions.


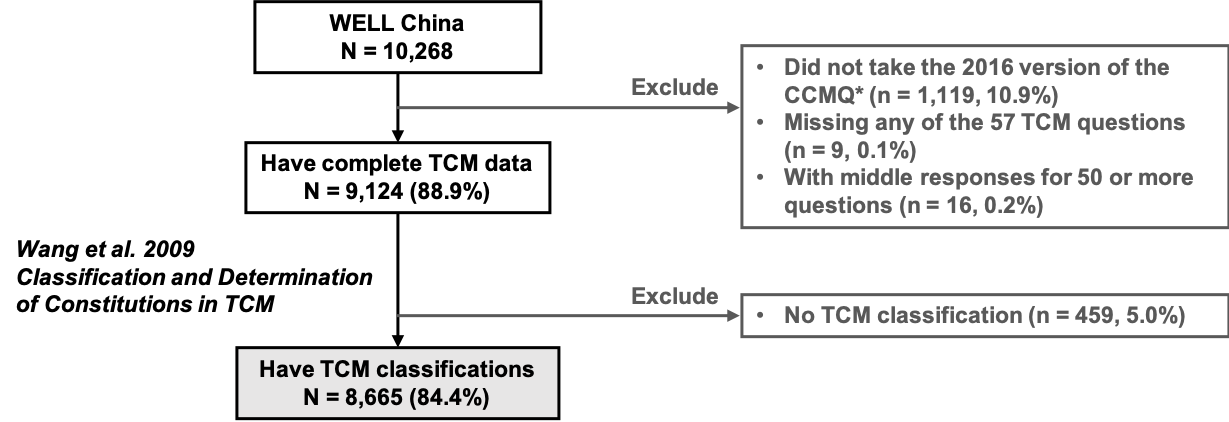


**Supplementary Figure S2. Inclusion criteria for analyzing the prevalences of TCM body constitutions among WELL China participants (N = 10,268).**

*CCMQ: The Constitution in Chinese Medicine Questionnaire. The 2016 version of CCMQ contains 54 items that are administered as 57 questions. Of the 1,119 participants, 764 completed an earlier version, and 355 did not respond to any TCM questionnaire.

**
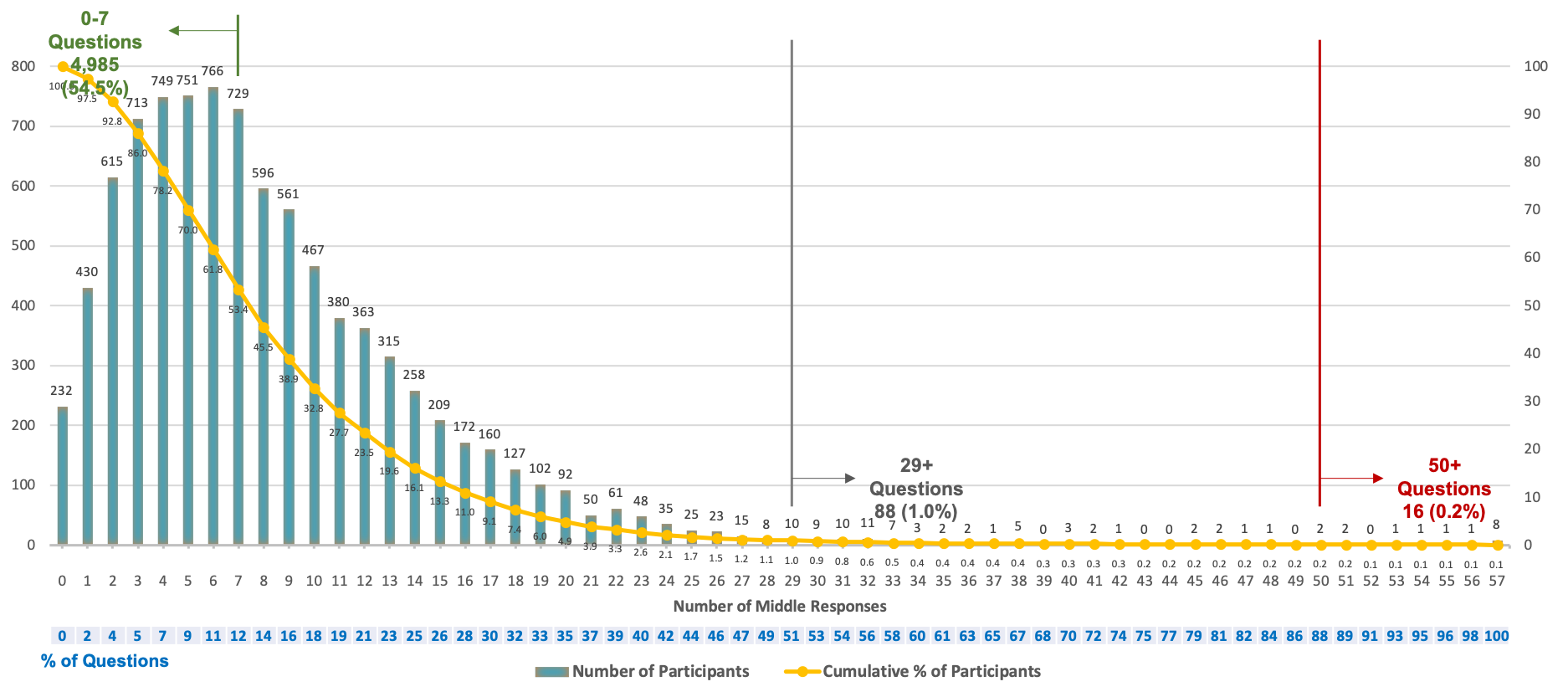
**

**Supplementary Figure S3. Distribution of number of middle responses on TCM questionnaire among participants with complete TCM data (N = 9,140).**

More than half of the participants (n = 4,985, 54.5%) selected the middle response for seven or fewer questions, and only 88 (1.0%) of participants selected the middle response for more than half of the questions (≥29 questions). The 16 (0.2%) participants who selected the middle response for 50 or more questions (88% of questions) were excluded from the analysis to minimize potential misclassification.

**
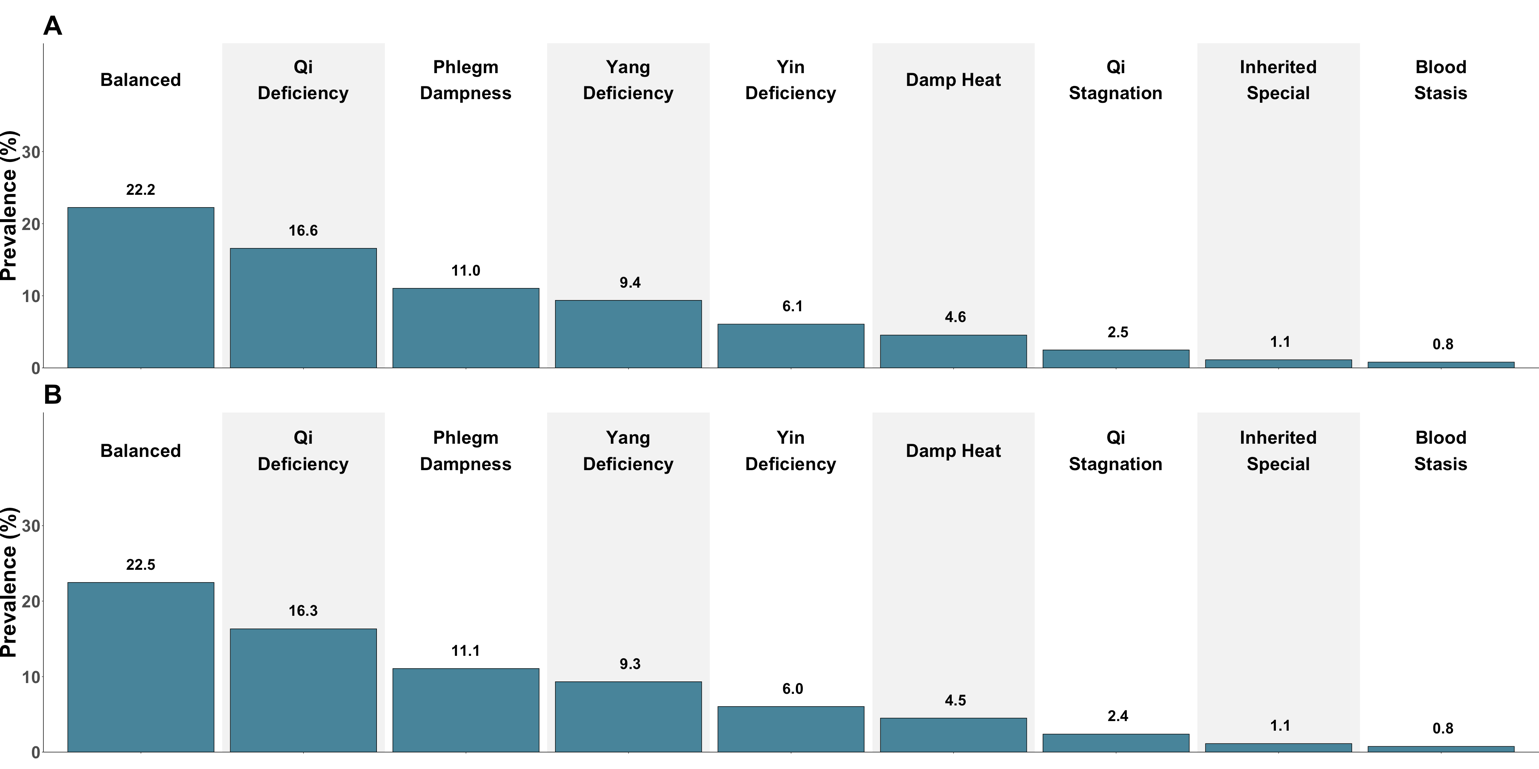
**

**Supplementary Figure S4. Sensitivity analysis of body constitution prevalence based on exclusion criteria for middle responses on the TCM questionnaire.**

Prevalences were calculated based on each participant’s primary body constitution.

**A.** Prevalence of body constitutions without excluding participants based on the number of middle responses (N = 8,681).

**B.** Prevalence of body constitutions excluding participants who selected the middle response for more than half of the questionnaire items (≥29 of 57 questions) (N = 8,593).


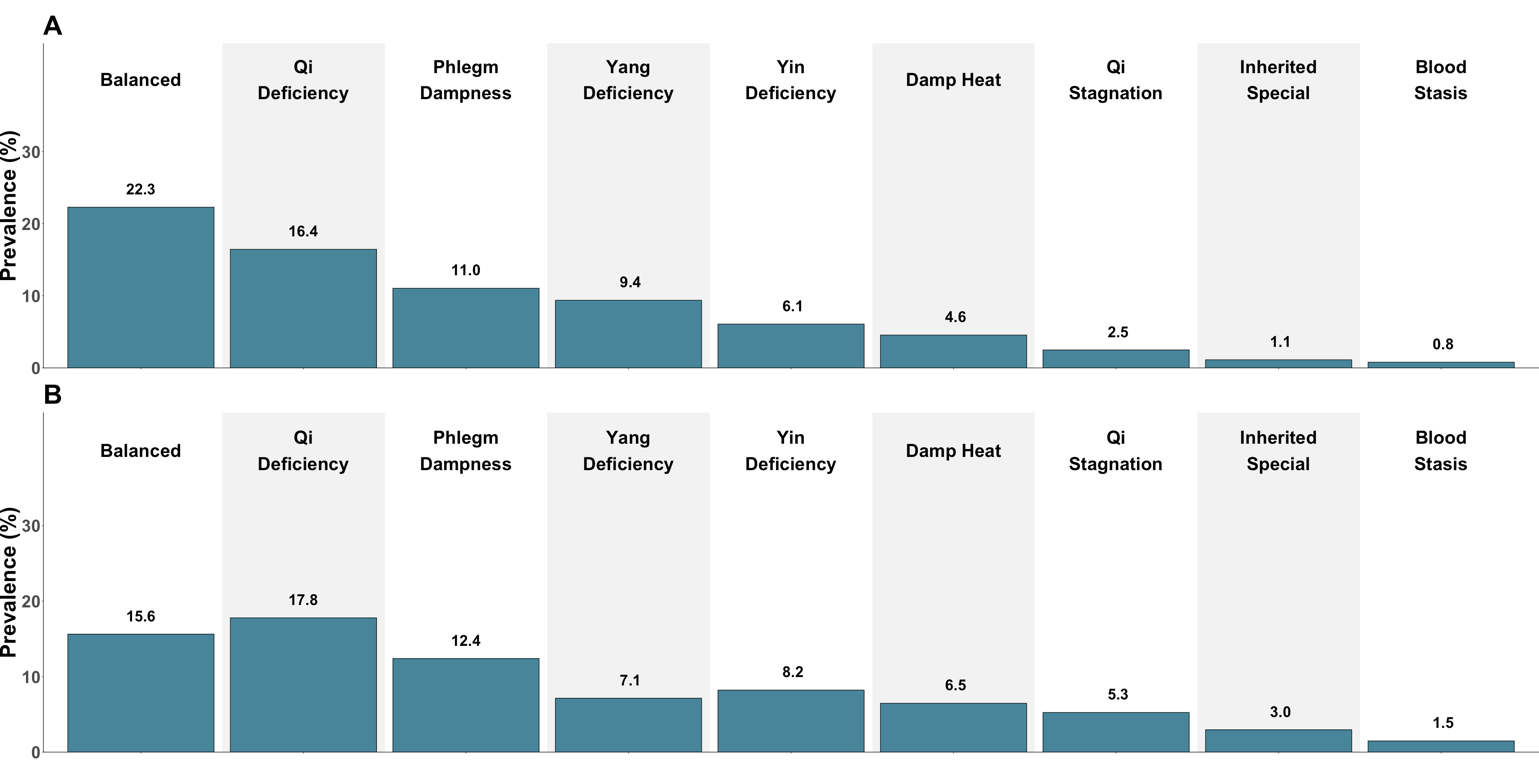


**Supplementary Figure S5. Comparison of the body constitution distributions between included participants and those excluded who completed an earlier version of the TCM questionnaire.**

Prevalences were calculated based on each participant’s primary body constitution.

**A.** Prevalence of body constitutions among included participants, who completed the 2016 version of the CCMQ (N =8,665).

**B.** Prevalence of body constitutions among participants who completed an earlier version of the CCMQ and had one or more body constitutions (N = 574). Among the 763 participants who completed an earlier version of the questionnaire, 574 had body constitutions, 168 had only unbalanced tendencies, and 21 had no classification.


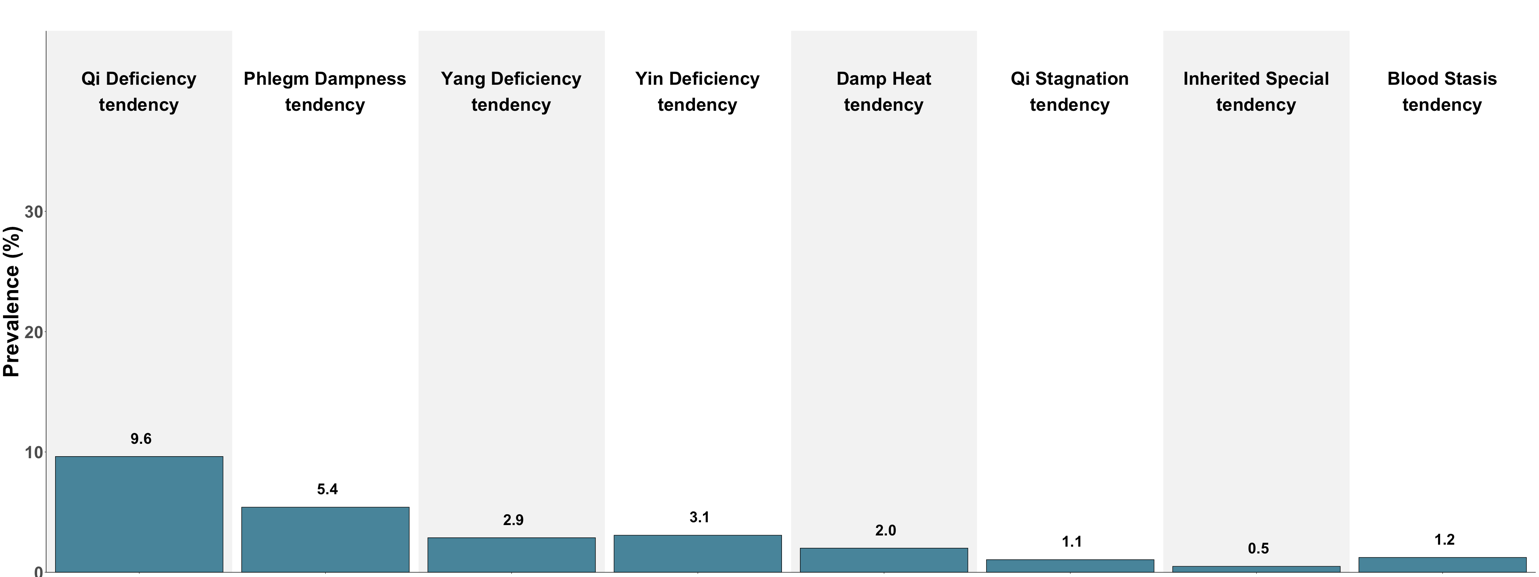


**Supplementary Figure S6. Prevalence of unbalanced tendencies (N = 8,665).**

Prevalences were calculated based on each participant’s primary unbalanced tendency.

**
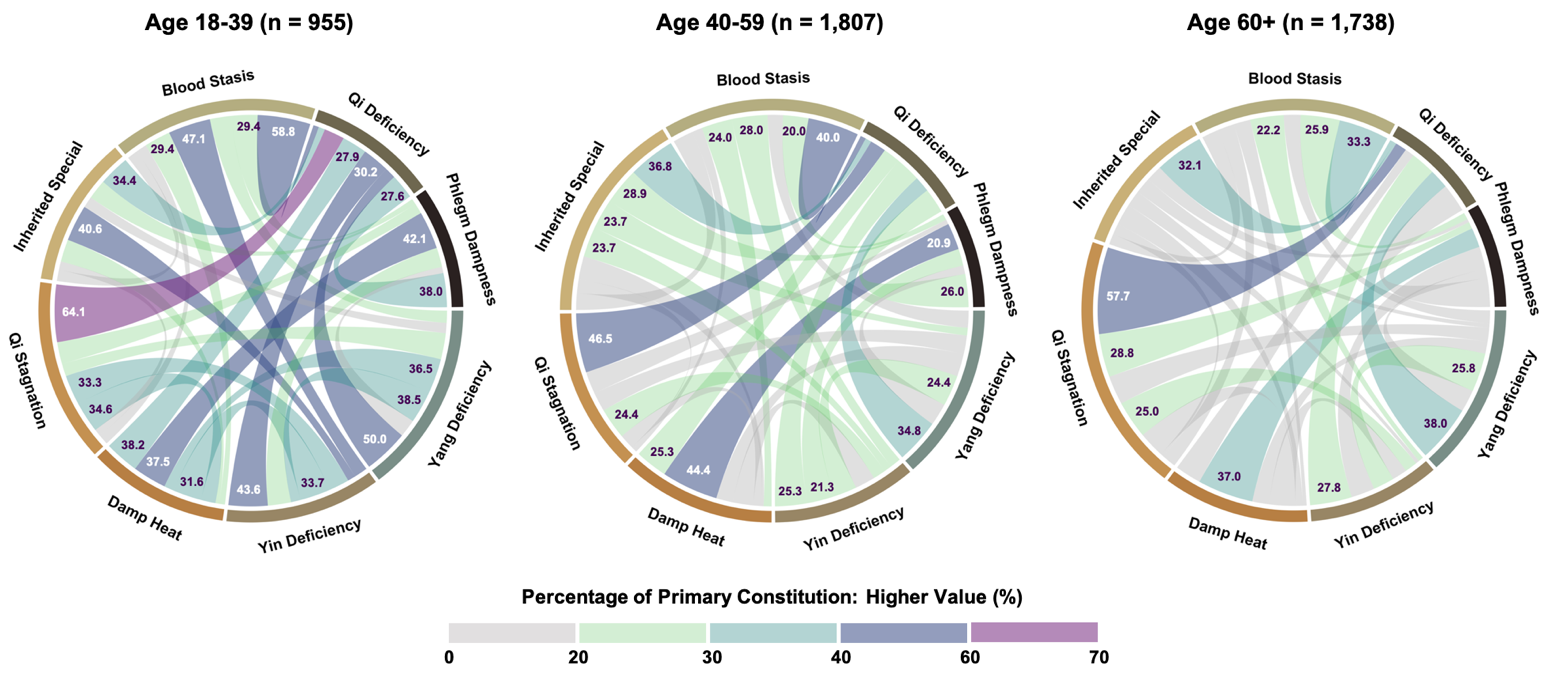
**

**Supplementary Figure S7. Percentage of coexisting body constitutions by primary constitution by age among participants with unbalanced constitutions (N = 4,500).**

Each segment around the circle represents one of the eight unbalanced constitutions. Chords connecting two segments indicates the coexistence of two constitutions. The width of each chord represents the percentage of participants with a coexisting body constitution, calculated within participants with the specified primary constitution. The width of the two ends of a chord may differ, as the percentage of participants with constitution A who also have constitution B is not necessarily equal to the percentage of participants with constitution B who also have constitution A. Chord color is based on the higher of the two percentages, with darker shades indicating higher values.

**Supplementary Table S1. The Constitution in Chinese Medicine Questionnaire (CCMQ) 2016, 54-item version.**

| The next questions are about your body constitution or body type. Please choose the most suitable response based on your actual situation or experience in the past year. If you are unsure of the answer to a specific question, choose the answer that is most similar to your actual situation.   For each question, please check one box. | | | | | | |
| --- | --- | --- | --- | --- | --- | --- |
| **No** | **In the past year…** | **Never** | **Rarely** | **Sometimes** | **Fairly often** | **All the time** |
| 1 | Were you full of energy? | □ | □ | □ | □ | □ |
| 2 | Did you feel lethargic or get tired easily? | □ | □ | □ | □ | □ |
| 3 | Did you prefer being quiet and not talking much? | □ | □ | □ | □ | □ |
| 4 | Did you handle situations with optimism and make adjustment when things did not go well? | □ | □ | □ | □ | □ |
| 5 | When you spoke, was your voice low and weak? | □ | □ | □ | □ | □ |
| 6 | Did you have shortness of breath or panting after climbing 3 flights of stairs or talking too much? | □ | □ | □ | □ | □ |
| 7 | Did you have a good appetite? | □ | □ | □ | □ | □ |
| 8 | Did you feel gloomy and low-spirited, lacking interest in work or life? | □ | □ | □ | □ | □ |
| 9 | Did you feel pessimistic easily? | □ | □ | □ | □ | □ |
| 10 | Did you often feel sentimental (if you watch a sad movie, you are affected emotionally more) or sigh for no apparent reason? | □ | □ | □ | □ | □ |
| 11 | Were you easily scared or frightened? | □ | □ | □ | □ | □ |
| 12 | Did you easily get nervous or anxious? | □ | □ | □ | □ | □ |
| 13 | Did you feel you are sensitive and suspicious (e.g., often thinking someone is speaking ill of you behind your back)? | □ | □ | □ | □ | □ |
| 14 | Did you have a dull complexion? | □ | □ | □ | □ | □ |
| 15 | Did you have swollen upper eyelids? | □ | □ | □ | □ | □ |
| 16 | Did your eyes feel dry and uncomfortable? | □ | □ | □ | □ | □ |
| 17 | Did your face feel flushed and you tend to have red cheeks? | □ | □ | □ | □ | □ |
| 18 | Were you less tolerant of cold compared to others (were you less comfortable when you are near the freezer section in supermarkets, or did you avoid using air conditioning in the summer)? | □ | □ | □ | □ | □ |
| 19 | Did you feel less tolerant of cold and avoid going near refrigerated food sections in the supermarkets? | □ | □ | □ | □ | □ |
| 20 | Did you catch colds more easily than others? | □ | □ | □ | □ | □ |
| 21-1 | Were you allergic to any particular foods (such as nuts, seafood, fish, dairy products, bean products, chickens, ducks, beef and mutton, alcohol, peaches, pears and other fruits etc.)? | □ | □ | □ | □ | □ |
| 21-2 | Were you allergic to certain things, particles, or particular odors when you inhaled them (such as pollen, catkins, dust, insects, mites, cold air, animal dander, lampblack, any kinds of spices, vehicle exhaust, gas, cigarettes, etc.)? | □ | □ | □ | □ | □ |
| 21-3 | Were you allergic to any particular physical or airborne things when you were exposed to them (such as cosmetics, hair dyes, paints, cold air, hot air, ultraviolet light (UV), detergent, soap, chemical products, plastic, and necklace, bracelet, watchband, rings, earrings and other metal accessories, etc.)? | □ | □ | □ | □ | □ |
| 21-4 | In addition to the factors mentioned above, did you have any allergic experience caused by other factors? (known or unknown factors) | □ | □ | □ | □ | □ |
| 22 | Did you forget things more easily compared to people your age? | □ | □ | □ | □ | □ |
| 23 | Did the color of your lips look darker? | □ | □ | □ | □ | □ |
| 24 | Did your palms or the soles of your feet often feel hot? | □ | □ | □ | □ | □ |
| 25 | Did you have an oily forehead? | □ | □ | □ | □ | □ |
| 26 | Did you have dry skin? | □ | □ | □ | □ | □ |
| 27 | Did you sweat easily with slight physical activity? | □ | □ | □ | □ | □ |
| 28 | Did you feel uncomfortable (stomach pain or diarrhea) when you had cold drinks or cold foods? | □ | □ | □ | □ | □ |
| 29 | Did green or purple bruises appear on your skin for no reason? | □ | □ | □ | □ | □ |
| 30 | Did your skin turn red and show raised red traces when scratched? | □ | □ | □ | □ | □ |
| 31 | Did you have visible capillary (thread) veins on your cheeks? | □ | □ | □ | □ | □ |
| 32 | Did you have dark spots appear on your face? | □ | □ | □ | □ | □ |
| 33 | Did your nose or your face feel greasy, oily, or shiny? | □ | □ | □ | □ | □ |
| 34 | Was your weight in the normal range, and you have moderate body size and shape? | □ | □ | □ | □ | □ |
| 35 | Did you feel sensitive to cold temperatures? | □ | □ | □ | □ | □ |
| 36 | Did you tend to wear more clothing than others; even during hot summer days, you did not wear short sleeves or shorts? | □ | □ | □ | □ | □ |
| 37 | Did your hair become oily easily? | □ | □ | □ | □ | □ |
| 38 | Did you have a bitter taste in your mouth or bad breath? | □ | □ | □ | □ | □ |
| 39 | Did you have dry lips or dry mouth and throat, and often feel like you had to drink water? | □ | □ | □ | □ | □ |
| 40-1 | For male participants: Did you feel distention, bloating and pain around your ribcage? | □ | □ | □ | □ | □ |
| 40-2 | For female participants: Did you often feel distention, bloating and pain in your breasts or around your ribcage? | □ | □ | □ | □ | □ |
| 41 | Did you get acne or mouth ulcers easily? | □ | □ | □ | □ | □ |
| 42 | Did you get constipated easily or have dry, hard stools? | □ | □ | □ | □ | □ |
| 43 | Did your hands or feet feel cold? | □ | □ | □ | □ | □ |
| 44 | Did you have allergic experiences? | □ | □ | □ | □ | □ |
| 45 | Were the veins under your tongue dark purple? | □ | □ | □ | □ | □ |
| 46 | Did you have regular bowel movements? | □ | □ | □ | □ | □ |
| 47 | Did you sleep well? | □ | □ | □ | □ | □ |
| 48 | Did people tell you that you snore while sleeping | □ | □ | □ | □ | □ |
| 49 | Were you able to adapt to changes in the external natural environment (changing seasons or weather) or geographical environment (high altitude)? | □ | □ | □ | □ | □ |
| 50 | Did you feel cold in your abdomen, back, lower back or knees? | □ | □ | □ | □ | □ |
| 51 | Did you have a chubby body type with extra fat around the waist or abdomen? | □ | □ | □ | □ | □ |
| 52 | Did you pass sticky stools and/or feel that your bowel movement was incomplete (e.g., stools that stick to the toilet bowl and not flush away easily)? | □ | □ | □ | □ | □ |
| 53 | Did you feel your limbs were heavy or uneasy to move? | □ | □ | □ | □ | □ |
| 54-1 | For male participants: Was your scrotal area damp? | □ | □ | □ | □ | □ |
| 54-2 | For female participants: Was your vaginal discharge yellowish? | □ | □ | □ | □ | □ |

**Supplementary Table S2. Comparison of demographic characteristics between participants included and excluded from the analysis (N = 10,268).**

| Characteristics | Total | Included | Excluded |  |
| --- | --- | --- | --- | --- |
|  | **n (%)** | **n (%)** | **n (%)** | ***p* value** |
| All | 10268 (100) | 8665 (84.4) | 1603 (15.6) |  |
| Age |  |  |  |  |
| 18–29 | 590 (5.7) | 467 (5.4) | 123 (7.7) | 0.001 |
| 30–49 | 2831 (27.6) | 2374 (27.4) | 457 (28.5) |  |
| 50–59 | 2627 (25.6) | 2233 (25.8) | 394 (24.6) |  |
| 60–69 | 3020 (29.4) | 2588 (29.9) | 432 (26.9) |  |
| 70+ | 1200 (11.7) | 1003 (11.6) | 197 (12.3) |  |
| Sex |  |  |  |  |
| Male | 4081 (39.7) | 3383 (39.0) | 698 (43.5) | <0.001 |
| Female | 6187 (60.3) | 5282 (61.0) | 905 (56.5) |  |
| Education |  |  |  |  |
| Elementary school and under | 2121 (21.3) | 1669 (19.3) | 452 (34.8) | <0.001 |
| Middle school | 3074 (30.9) | 2751 (31.7) | 323 (24.9) |  |
| High school | 2304 (23.1) | 2062 (23.8) | 242 (18.7) |  |
| College and above | 2463 (24.7) | 2183 (25.2) | 280 (21.6) |  |
| Annual Income (USD) |  |  |  |  |
| <$3104 | 1396 (14) | 1034 (11.9) | 362 (27.9) | <0.001 |
| $3104–$7760 | 4901 (49.2) | 4417 (51) | 484 (37.3) |  |
| $7760–$12416 | 2204 (22.1) | 1978 (22.8) | 226 (17.4) |  |
| $12416+ | 1459 (14.6) | 1234 (14.2) | 225 (17.3) |  |
| Work Status |  |  |  |  |
| Working | 3036 (30.5) | 2533 (29.2) | 503 (38.8) | <0.001 |
| Others | 2203 (22.1) | 1862 (21.5) | 341 (26.3) |  |
| Retired | 4723 (47.4) | 4270 (49.3) | 453 (34.9) |  |
| Marital Status |  |  |  |  |
| Married | 8692 (87.3) | 7551 (87.1) | 1141 (88.0) | 0.44 |
| Others | 715 (7.2) | 633 (7.3) | 82 (6.3) |  |
| Single | 555 (5.6) | 481 (5.6) | 74 (5.7) |  |
| Smoking Status |  |  |  |  |
| Never | 7374 (74.1) | 6456 (74.5) | 918 (71.0) | 0.02 |
| Former | 718 (7.2) | 620 (7.2) | 98 (7.6) |  |
| Current | 1865 (18.7) | 1588 (18.3) | 277 (21.4) |  |
| Alcohol Drinking Status |  |  |  |  |
| Never | 5385 (54.0) | 4677 (54.0) | 708 (54.0) | 0.17 |
| Former | 199 (2.0) | 169 (2.0) | 30 (2.3) |  |
| Current occasionally | 2457 (24.6) | 2160 (24.9) | 297 (22.7) |  |
| Current frequent | 1934 (19.4) | 1659 (19.1) | 275 (21.0) |  |
| Body Mass Index (BMI) |  |  |  |  |
| <18.5 (Underweight) | 394 (3.9) | 328 (3.8) | 66 (4.2) | 0.20 |
| 18.5–23.9 (Normal) | 5355 (52.4) | 4503 (52.0) | 852 (54.4) |  |
| 24.0–27.9 (Overweight) | 3507 (34.3) | 2995 (34.6) | 512 (32.7) |  |
| 28+ (Obese) | 961 (9.4) | 826 (9.5) | 135 (8.6) |  |

**Supplementary Table S3. Proportion with composite constitutions by age group and primary constitution among participants with unbalanced constitutions (N = 4,500).**

|  | Composite constitutions | | | |
| --- | --- | --- | --- | --- |
| Age group | **18–39 (n = 955)** | **40–59 (n = 1807)** | **60+ (n = 1738)** |  |
| Primary constitution (n) | **n (%)** | **n (%)** | **n (%)** | ***p* value*** |
| All unbalanced constitutions (4500) | 643 (67.3) | 934 (51.7) | 811 (46.7) | < 0.001 |
|  |  |  |  |  |
| Qi Deficiency (1425) | 197 (64.0) | 263 (47.0) | 232 (41.7) | < 0.001 |
| Qi Deficiency (1425) | 106 (62.0) | 185 (44.5) | 126 (34.1) | < 0.001 |
| Yang Deficiency (813) | 74 (77.1) | 183 (61.2) | 248 (59.3) | 0.006 |
| Phlegm Dampness (957) | 73 (72.3) | 109 (49.3) | 96 (46.8) | < 0.001 |
| Damp Heat (395) | 95 (62.5) | 94 (58.0) | 42 (51.9) | 0.12 |
| Yang Deficiency (813) | 60 (76.9) | 57 (66.3) | 39 (75.0) | 0.66 |
| Inherited Special (98) | 23 (71.9) | 27 (71.1) | 12 (42.9) | 0.02 |
| Yin Deficiency (527) | 15 (88.2) | 16 (64.0) | 16 (59.3) | 0.06 |

*Chi-squared tests for trends evaluated the percentages of composite constitutions across age groups, with *p* values < 0.006 (Bonferroni-corrected significance level) considered statistically significant.

**Supplementary Table S4. Adjusted prevalence of primary body constitutions by demographic and lifestyle characteristics (N = 8,665).**

| Body constitution | Subgroup | N (%) | Crude prevalence, n (%) | Adjusted prevalence (%)^1^ | aPR (95% CI)^1^ | *p* value |
| --- | --- | --- | --- | --- | --- | --- |
| Education | |  |  |  |  |  |
| Balanced | Elementary school and under | 1669 (19.3) | 395 (23.7) | 21.1 | 1.0 (Reference) |  |
|  | Middle school | 2751 (31.7) | 654 (23.8) | 21.1 | 1.00 (0.90, 1.12) | 0.96 |
|  | High school | 2062 (23.8) | 494 (24.0) | 22.2 | 1.05 (0.94, 1.19) | 0.39 |
|  | College and above | 2183 (25.2) | 388 (17.8) | 18.4 | 0.87 (0.76, 1.00) | 0.06 |
| Qi Deficiency | Elementary school and under | 1669 (19.3) | 286 (17.1) | 18.8 | 1.0 (Reference) |  |
|  | Middle school | 2751 (31.7) | 442 (16.1) | 17.5 | 0.93 (0.81, 1.07) | 0.33 |
|  | High school | 2062 (23.8) | 283 (13.7) | 14.6 | 0.77 (0.66, 0.90) | 0.001 |
|  | College and above | 2183 (25.2) | 414 (19.0) | 18.4 | 0.98 (0.84, 1.14) | 0.79 |
| Phlegm Dampness | Elementary school and under | 1669 (19.3) | 181 (10.8) | 10.9 | 1.0 (Reference) |  |
|  | Middle school | 2751 (31.7) | 288 (10.5) | 9.6 | 0.88 (0.74, 1.05) | 0.15 |
|  | High school | 2062 (23.8) | 230 (11.2) | 10.1 | 0.93 (0.77, 1.12) | 0.44 |
|  | College and above | 2183 (25.2) | 258 (11.8) | 10.9 | 1.00 (0.83, 1.21) | 0.98 |
| Yang Deficiency | Elementary school and under | 1669 (19.3) | 171 (10.2) | 6.6 | 1.0 (Reference) |  |
|  | Middle school | 2751 (31.7) | 262 (9.5) | 7.1 | 1.07 (0.89, 1.29) | 0.46 |
|  | High school | 2062 (23.8) | 195 (9.5) | 7.5 | 1.14 (0.93, 1.39) | 0.2 |
|  | College and above | 2183 (25.2) | 185 (8.5) | 8.0 | 1.21 (0.98, 1.50) | 0.08 |
| Yin Deficiency | Elementary school and under | 1669 (19.3) | 94 (5.6) | 4.7 | 1.0 (Reference) |  |
|  | Middle school | 2751 (31.7) | 158 (5.7) | 5.1 | 1.10 (0.85, 1.41) | 0.47 |
|  | High school | 2062 (23.8) | 124 (6.0) | 5.3 | 1.14 (0.87, 1.49) | 0.35 |
|  | College and above | 2183 (25.2) | 151 (6.9) | 6.0 | 1.30 (0.98, 1.72) | 0.07 |
| Damp Heat | Elementary school and under | 1669 (19.3) | 41 (2.5) | 3.1 | 1.0 (Reference) |  |
|  | Middle school | 2751 (31.7) | 88 (3.2) | 3.4 | 1.09 (0.76, 1.57) | 0.65 |
|  | High school | 2062 (23.8) | 92 (4.5) | 4.0 | 1.28 (0.88, 1.86) | 0.19 |
|  | College and above | 2183 (25.2) | 174 (8.0) | 5.5 | 1.75 (1.22, 2.51) | 0.002 |
| Qi Stagnation | Elementary school and under | 1669 (19.3) | 41 (2.5) | 3.0 | 1.0 (Reference) |  |
|  | Middle school | 2751 (31.7) | 46 (1.7) | 2.0 | 0.66 (0.44, 1.01) | 0.06 |
|  | High school | 2062 (23.8) | 42 (2.0) | 2.0 | 0.67 (0.43, 1.04) | 0.08 |
|  | College and above | 2183 (25.2) | 87 (4.0) | 2.6 | 0.88 (0.57, 1.36) | 0.57 |
| Inherited Special | Elementary school and under | 1669 (19.3) | 4 (0.2) | 0.2 | 1.0 (Reference) |  |
|  | Middle school | 2751 (31.7) | 22 (0.8) | 0.8 | 3.40 (1.17, 9.87) | 0.02 |
|  | High school | 2062 (23.8) | 33 (1.6) | 1.6 | 6.44 (2.29, 18.08) | < 0.001 |
|  | College and above | 2183 (25.2) | 39 (1.8) | 1.3 | 5.56 (1.90, 16.28) | 0.002 |
| Blood Stasis | Elementary school and under | 1669 (19.3) | 15 (0.9) | 0.7 | 1.0 (Reference) |  |
|  | Middle school | 2751 (31.7) | 20 (0.7) | 0.6 | 0.89 (0.45, 1.76) | 0.73 |
|  | High school | 2062 (23.8) | 14 (0.7) | 0.5 | 0.80 (0.38, 1.67) | 0.55 |
|  | College and above | 2183 (25.2) | 20 (0.9) | 0.7 | 0.99 (0.47, 2.11) | 0.99 |
| Annual Income (USD) | |  |  |  |  |  |
| Balanced | <$3104 | 1034 (11.9) | 184 (17.8) | 16.8 | 1.0 (Reference) |  |
|  | $3104–$7760 | 4417 (51) | 1037 (23.5) | 21.1 | 1.26 (1.09, 1.45) | 0.001 |
|  | $7760–$12416 | 1978 (22.8) | 461 (23.3) | 21.6 | 1.29 (1.11, 1.51) | 0.001 |
|  | $12416+ | 1234 (14.2) | 249 (20.2) | 19.7 | 1.18 (0.99, 1.41) | 0.07 |
| Qi Deficiency | <$3104 | 1034 (11.9) | 219 (21.2) | 21.9 | 1.0 (Reference) |  |
|  | $3104–$7760 | 4417 (51) | 692 (15.7) | 16.9 | 0.77 (0.67, 0.88) | < 0.001 |
|  | $7760–$12416 | 1978 (22.8) | 320 (16.2) | 16.8 | 0.76 (0.65, 0.89) | < 0.001 |
|  | $12416+ | 1234 (14.2) | 193 (15.6) | 15.8 | 0.72 (0.60, 0.87) | < 0.001 |
| Phlegm Dampness | <$3104 | 1034 (11.9) | 106 (10.3) | 10.7 | 1.0 (Reference) |  |
|  | $3104–$7760 | 4417 (51) | 427 (9.7) | 9.8 | 0.92 (0.75, 1.12) | 0.39 |
|  | $7760–$12416 | 1978 (22.8) | 225 (11.4) | 9.9 | 0.93 (0.74, 1.15) | 0.49 |
|  | $12416+ | 1234 (14.2) | 199 (16.1) | 13.0 | 1.22 (0.98, 1.53) | 0.08 |
| Yang Deficiency | <$3104 | 1034 (11.9) | 111 (10.7) | 8.0 | 1.0 (Reference) |  |
|  | $3104–$7760 | 4417 (51) | 474 (10.7) | 7.6 | 0.95 (0.78, 1.16) | 0.63 |
|  | $7760–$12416 | 1978 (22.8) | 158 (8.0) | 7.4 | 0.92 (0.73, 1.17) | 0.5 |
|  | $12416+ | 1234 (14.2) | 70 (5.7) | 6.0 | 0.75 (0.56, 1.01) | 0.06 |
| Yin Deficiency | <$3104 | 1034 (11.9) | 73 (7.1) | 5.7 | 1.0 (Reference) |  |
|  | $3104–$7760 | 4417 (51) | 284 (6.4) | 5.4 | 0.94 (0.74, 1.21) | 0.65 |
|  | $7760–$12416 | 1978 (22.8) | 105 (5.3) | 5.2 | 0.91 (0.68, 1.21) | 0.5 |
|  | $12416+ | 1234 (14.2) | 65 (5.3) | 5.3 | 0.93 (0.67, 1.30) | 0.66 |
| Damp Heat | <$3104 | 1034 (11.9) | 39 (3.8) | 3.8 | 1.0 (Reference) |  |
|  | $3104–$7760 | 4417 (51) | 138 (3.1) | 3.6 | 0.94 (0.66, 1.33) | 0.72 |
|  | $7760–$12416 | 1978 (22.8) | 112 (5.7) | 4.7 | 1.23 (0.86, 1.76) | 0.25 |
|  | $12416+ | 1234 (14.2) | 106 (8.6) | 5.3 | 1.40 (0.96, 2.03) | 0.08 |
| Qi Stagnation | <$3104 | 1034 (11.9) | 36 (3.5) | 3.0 | 1.0 (Reference) |  |
|  | $3104–$7760 | 4417 (51) | 99 (2.2) | 2.4 | 0.79 (0.54, 1.16) | 0.23 |
|  | $7760–$12416 | 1978 (22.8) | 53 (2.7) | 2.4 | 0.80 (0.52, 1.23) | 0.31 |
|  | $12416+ | 1234 (14.2) | 28 (2.3) | 1.8 | 0.60 (0.36, 0.99) | 0.05 |
| Inherited Special | <$3104 | 1034 (11.9) | 15 (1.5) | 1.1 | 1.0 (Reference) |  |
|  | $3104–$7760 | 4417 (51) | 31 (0.7) | 0.6 | 0.57 (0.30, 1.07) | 0.08 |
|  | $7760–$12416 | 1978 (22.8) | 35 (1.8) | 1.6 | 1.48 (0.80, 2.74) | 0.22 |
|  | $12416+ | 1234 (14.2) | 17 (1.4) | 1.3 | 1.16 (0.58, 2.34) | 0.68 |
| Blood Stasis | <$3104 | 1034 (11.9) | 9 (0.9) | 0.6 | 1.0 (Reference) |  |
|  | $3104–$7760 | 4417 (51) | 36 (0.8) | 0.6 | 0.95 (0.46, 1.97) | 0.89 |
|  | $7760–$12416 | 1978 (22.8) | 12 (0.6) | 0.5 | 0.82 (0.34, 2.03) | 0.68 |
|  | $12416+ | 1234 (14.2) | 12 (1.0) | 0.9 | 1.35 (0.51, 3.54) | 0.55 |
| Work Status | |  |  |  |  |  |
| Balanced | Working | 2533 (29.2) | 532 (21.0) | 21.0 | 1.0 (Reference) |  |
|  | Others | 1862 (21.5) | 362 (19.4) | 19.9 | 0.95 (0.84, 1.07) | 0.38 |
|  | Retired | 4270 (49.3) | 1037 (24.3) | 20.4 | 0.97 (0.85, 1.11) | 0.65 |
| Qi Deficiency | Working | 2533 (29.2) | 415 (16.4) | 16.6 | 1.0 (Reference) |  |
|  | Others | 1862 (21.5) | 351 (18.9) | 18.9 | 1.13 (1.00, 1.29) | 0.06 |
|  | Retired | 4270 (49.3) | 659 (15.4) | 17.1 | 1.03 (0.86, 1.22) | 0.78 |
| Phlegm Dampness | Working | 2533 (29.2) | 340 (13.4) | 11.1 | 1.0 (Reference) |  |
|  | Others | 1862 (21.5) | 190 (10.2) | 9.2 | 0.83 (0.71, 0.99) | 0.03 |
|  | Retired | 4270 (49.3) | 427 (10.0) | 10.5 | 0.95 (0.77, 1.17) | 0.63 |
| Yang Deficiency | Working | 2533 (29.2) | 168 (6.6) | 6.7 | 1.0 (Reference) |  |
|  | Others | 1862 (21.5) | 153 (8.2) | 7.6 | 1.13 (0.91, 1.39) | 0.27 |
|  | Retired | 4270 (49.3) | 492 (11.5) | 7.7 | 1.15 (0.90, 1.47) | 0.27 |
| Yin Deficiency | Working | 2533 (29.2) | 137 (5.4) | 4.9 | 1.0 (Reference) |  |
|  | Others | 1862 (21.5) | 120 (6.4) | 5.2 | 1.07 (0.85, 1.36) | 0.56 |
|  | Retired | 4270 (49.3) | 270 (6.3) | 5.9 | 1.21 (0.90, 1.62) | 0.22 |
| Damp Heat | Working | 2533 (29.2) | 178 (7.0) | 4.8 | 1.0 (Reference) |  |
|  | Others | 1862 (21.5) | 124 (6.7) | 5.0 | 1.03 (0.82, 1.28) | 0.82 |
|  | Retired | 4270 (49.3) | 93 (2.2) | 3.3 | 0.69 (0.50, 0.96) | 0.03 |
| Qi Stagnation | Working | 2533 (29.2) | 82 (3.2) | 2.4 | 1.0 (Reference) |  |
|  | Others | 1862 (21.5) | 66 (3.5) | 2.4 | 0.99 (0.72, 1.37) | 0.96 |
|  | Retired | 4270 (49.3) | 68 (1.6) | 2.3 | 0.94 (0.58, 1.52) | 0.79 |
| Inherited Special | Working | 2533 (29.2) | 32 (1.3) | 0.9 | 1.0 (Reference) |  |
|  | Others | 1862 (21.5) | 29 (1.6) | 1.0 | 1.09 (0.66, 1.80) | 0.74 |
|  | Retired | 4270 (49.3) | 37 (0.9) | 1.1 | 1.20 (0.55, 2.60) | 0.65 |
| Blood Stasis | Working | 2533 (29.2) | 20 (0.8) | 0.7 | 1.0 (Reference) |  |
|  | Others | 1862 (21.5) | 18 (1.0) | 0.8 | 1.10 (0.57, 2.10) | 0.78 |
|  | Retired | 4270 (49.3) | 31 (0.7) | 0.5 | 0.74 (0.31, 1.79) | 0.51 |
| Marital Status | |  |  |  |  |  |
| Balanced | Married | 7551 (87.1) | 1741 (23.1) | 21.3 | 1.0 (Reference) |  |
|  | Others | 633 (7.3) | 124 (19.6) | 18.2 | 0.85 (0.72, 1.00) | 0.05 |
|  | Single | 481 (5.6) | 66 (13.7) | 17.4 | 0.81 (0.61, 1.08) | 0.15 |
| Qi Deficiency | Married | 7551 (87.1) | 1203 (15.9) | 17.0 | 1.0 (Reference) |  |
|  | Others | 633 (7.3) | 110 (17.4) | 18.6 | 1.09 (0.91, 1.31) | 0.33 |
|  | Single | 481 (5.6) | 112 (23.3) | 18.9 | 1.11 (0.87, 1.41) | 0.39 |
| Phlegm Dampness | Married | 7551 (87.1) | 851 (11.3) | 10.6 | 1.0 (Reference) |  |
|  | Others | 633 (7.3) | 61 (9.6) | 10.6 | 1.01 (0.79, 1.29) | 0.96 |
|  | Single | 481 (5.6) | 45 (9.4) | 9.3 | 0.88 (0.59, 1.32) | 0.55 |
| Yang Deficiency | Married | 7551 (87.1) | 701 (9.3) | 7.4 | 1.0 (Reference) |  |
|  | Others | 633 (7.3) | 83 (13.1) | 8.2 | 1.12 (0.90, 1.38) | 0.31 |
|  | Single | 481 (5.6) | 29 (6.0) | 7.2 | 0.97 (0.63, 1.51) | 0.91 |
| Yin Deficiency | Married | 7551 (87.1) | 454 (6.0) | 5.2 | 1.0 (Reference) |  |
|  | Others | 633 (7.3) | 36 (5.7) | 4.3 | 0.82 (0.59, 1.15) | 0.26 |
|  | Single | 481 (5.6) | 37 (7.7) | 6.6 | 1.26 (0.81, 1.98) | 0.3 |
| Damp Heat | Married | 7551 (87.1) | 332 (4.4) | 4.1 | 1.0 (Reference) |  |
|  | Others | 633 (7.3) | 20 (3.2) | 3.8 | 0.93 (0.60, 1.44) | 0.74 |
|  | Single | 481 (5.6) | 43 (8.9) | 4.4 | 1.08 (0.70, 1.66) | 0.73 |
| Qi Stagnation | Married | 7551 (87.1) | 156 (2.1) | 2.0 | 1.0 (Reference) |  |
|  | Others | 633 (7.3) | 30 (4.7) | 4.9 | 2.40 (1.62, 3.57) | < 0.001 |
|  | Single | 481 (5.6) | 30 (6.2) | 2.8 | 1.37 (0.84, 2.23) | 0.2 |
| Inherited Special | Married | 7551 (87.1) | 80 (1.1) | 1.0 | 1.0 (Reference) |  |
|  | Others | 633 (7.3) | 3 (0.5) | 0.5 | 0.44 (0.14, 1.40) | 0.17 |
|  | Single | 481 (5.6) | 15 (3.1) | 1.3 | 1.27 (0.57, 2.82) | 0.56 |
| Blood Stasis | Married | 7551 (87.1) | 55 (0.7) | 0.5 | 1.0 (Reference) |  |
|  | Others | 633 (7.3) | 9 (1.4) | 0.9 | 1.70 (0.86, 3.39) | 0.13 |
|  | Single | 481 (5.6) | 5 (1.0) | 1.1 | 2.07 (0.44, 9.79) | 0.36 |
| Smoking Status | |  |  |  |  |  |
| Balanced | Never | 6456 (74.5) | 1425 (22.1) | 21.3 | 1.0 (Reference) |  |
|  | Former | 620 (7.2) | 145 (23.4) | 18.5 | 0.87 (0.74, 1.03) | 0.1 |
|  | Current | 1588 (18.3) | 360 (22.7) | 18.7 | 0.88 (0.77, 1.00) | 0.04 |
| Qi Deficiency | Never | 6456 (74.5) | 1069 (16.6) | 17.2 | 1.0 (Reference) |  |
|  | Former | 620 (7.2) | 89 (14.4) | 16.1 | 0.94 (0.76, 1.17) | 0.58 |
|  | Current | 1588 (18.3) | 267 (16.8) | 18.4 | 1.07 (0.92, 1.25) | 0.36 |
| Phlegm Dampness | Never | 6456 (74.5) | 581 (9.0) | 9.7 | 1.0 (Reference) |  |
|  | Former | 620 (7.2) | 96 (15.5) | 11.4 | 1.18 (0.94, 1.47) | 0.16 |
|  | Current | 1588 (18.3) | 280 (17.6) | 12.4 | 1.28 (1.08, 1.52) | 0.005 |
| Yang Deficiency | Never | 6456 (74.5) | 708 (11.0) | 7.9 | 1.0 (Reference) |  |
|  | Former | 620 (7.2) | 36 (5.8) | 6.9 | 0.88 (0.61, 1.27) | 0.49 |
|  | Current | 1588 (18.3) | 69 (4.3) | 6.1 | 0.77 (0.57, 1.04) | 0.09 |
| Yin Deficiency | Never | 6456 (74.5) | 434 (6.7) | 4.7 | 1.0 (Reference) |  |
|  | Former | 620 (7.2) | 34 (5.5) | 8.8 | 1.85 (1.26, 2.73) | 0.002 |
|  | Current | 1588 (18.3) | 59 (3.7) | 6.1 | 1.29 (0.91, 1.83) | 0.15 |
| Damp Heat | Never | 6456 (74.5) | 265 (4.1) | 4.2 | 1.0 (Reference) |  |
|  | Former | 620 (7.2) | 32 (5.2) | 4.3 | 1.01 (0.68, 1.50) | 0.95 |
|  | Current | 1588 (18.3) | 98 (6.2) | 3.9 | 0.92 (0.70, 1.21) | 0.53 |
| Qi Stagnation | Never | 6456 (74.5) | 178 (2.8) | 2.3 | 1.0 (Reference) |  |
|  | Former | 620 (7.2) | 11 (1.8) | 2.8 | 1.20 (0.59, 2.45) | 0.62 |
|  | Current | 1588 (18.3) | 27 (1.7) | 2.3 | 0.97 (0.56, 1.66) | 0.9 |
| Inherited Special | Never | 6456 (74.5) | 89 (1.4) | 1.3 | 1.0 (Reference) |  |
|  | Former | 620 (7.2) | 1 (0.2) | 0.2 | 0.17 (0.02, 1.23) | 0.08 |
|  | Current | 1588 (18.3) | 8 (0.5) | 0.6 | 0.45 (0.20, 0.99) | 0.05 |
| Blood Stasis | Never | 6456 (74.5) | 58 (0.9) | 0.6 | 1.0 (Reference) |  |
|  | Former | 620 (7.2) | 4 (0.6) | 1.0 | 1.81 (0.55, 5.93) | 0.33 |
|  | Current | 1588 (18.3) | 7 (0.4) | 0.7 | 1.31 (0.49, 3.50) | 0.59 |
| Alcohol Consumption Status | |  |  |  |  |  |
| Balanced | Never | 4677 (54) | 994 (21.3) | 19.7 | 1.0 (Reference) |  |
|  | Former | 169 (2) | 34 (20.1) | 17.1 | 0.87 (0.64, 1.18) | 0.37 |
|  | Current occasionally | 2160 (24.9) | 452 (20.9) | 19.9 | 1.01 (0.91, 1.11) | 0.89 |
|  | Current frequent | 1659 (19.1) | 451 (27.2) | 23.6 | 1.19 (1.07, 1.33) | 0.001 |
| Qi Deficiency | Never | 4677 (54) | 825 (17.6) | 18.9 | 1.0 (Reference) |  |
|  | Former | 169 (2) | 26 (15.4) | 16.8 | 0.89 (0.61, 1.28) | 0.52 |
|  | Current occasionally | 2160 (24.9) | 365 (16.9) | 17.5 | 0.93 (0.83, 1.04) | 0.19 |
|  | Current frequent | 1659 (19.1) | 209 (12.6) | 13.5 | 0.71 (0.61, 0.83) | < 0.001 |
| Phlegm Dampness | Never | 4677 (54) | 410 (8.8) | 9.3 | 1.0 (Reference) |  |
|  | Former | 169 (2) | 20 (11.8) | 9.9 | 1.07 (0.69, 1.64) | 0.77 |
|  | Current occasionally | 2160 (24.9) | 256 (11.9) | 10.9 | 1.18 (1.01, 1.37) | 0.03 |
|  | Current frequent | 1659 (19.1) | 271 (16.3) | 12.6 | 1.36 (1.16, 1.59) | < 0.001 |
| Yang Deficiency | Never | 4677 (54) | 553 (11.8) | 8.7 | 1.0 (Reference) |  |
|  | Former | 169 (2) | 15 (8.9) | 8.1 | 0.93 (0.57, 1.52) | 0.76 |
|  | Current occasionally | 2160 (24.9) | 171 (7.9) | 7.0 | 0.80 (0.68, 0.94) | 0.008 |
|  | Current frequent | 1659 (19.1) | 74 (4.5) | 4.7 | 0.54 (0.42, 0.69) | < 0.001 |
| Yin Deficiency | Never | 4677 (54) | 299 (6.4) | 5.0 | 1.0 (Reference) |  |
|  | Former | 169 (2) | 11 (6.5) | 7.2 | 1.44 (0.80, 2.59) | 0.23 |
|  | Current occasionally | 2160 (24.9) | 145 (6.7) | 6.0 | 1.20 (0.98, 1.45) | 0.07 |
|  | Current frequent | 1659 (19.1) | 72 (4.3) | 5.3 | 1.06 (0.81, 1.39) | 0.68 |
| Damp Heat | Never | 4677 (54) | 177 (3.8) | 3.9 | 1.0 (Reference) |  |
|  | Former | 169 (2) | 8 (4.7) | 5.0 | 1.29 (0.65, 2.55) | 0.47 |
|  | Current occasionally | 2160 (24.9) | 116 (5.4) | 4.2 | 1.08 (0.86, 1.36) | 0.5 |
|  | Current frequent | 1659 (19.1) | 94 (5.7) | 4.7 | 1.23 (0.95, 1.59) | 0.11 |
| Qi Stagnation | Never | 4677 (54) | 122 (2.6) | 2.4 | 1.0 (Reference) |  |
|  | Former | 169 (2) | 4 (2.4) | 3.3 | 1.42 (0.52, 3.88) | 0.5 |
|  | Current occasionally | 2160 (24.9) | 70 (3.2) | 2.8 | 1.20 (0.89, 1.61) | 0.23 |
|  | Current frequent | 1659 (19.1) | 20 (1.2) | 1.6 | 0.68 (0.41, 1.11) | 0.12 |
| Inherited Special | Never | 4677 (54) | 63 (1.3) | 1.2 | 1.0 (Reference) |  |
|  | Former | 169 (2) | 0 (0.0) | 0 | 0.00 (0.00, 0.00) | < 0.001 |
|  | Current occasionally | 2160 (24.9) | 29 (1.3) | 1.2 | 0.98 (0.62, 1.54) | 0.92 |
|  | Current frequent | 1659 (19.1) | 6 (0.4) | 0.5 | 0.38 (0.16, 0.93) | 0.03 |
| Blood Stasis | Never | 4677 (54) | 42 (0.9) | 0.6 | 1.0 (Reference) |  |
|  | Former | 169 (2) | 3 (1.8) | 1.9 | 3.03 (0.91, 10.05) | 0.07 |
|  | Current occasionally | 2160 (24.9) | 16 (0.7) | 0.6 | 0.95 (0.53, 1.70) | 0.85 |
|  | Current frequent | 1659 (19.1) | 8 (0.5) | 0.6 | 0.90 (0.41, 1.95) | 0.79 |
| BMI | |  |  |  |  |  |
| Balanced | 18.5–23.9 (Normal) | 4503 (52) | 1163 (25.8) | 24.1 | 1.0 (Reference) |  |
|  | <18.5 (Underweight) | 328 (3.8) | 65 (19.8) | 19.7 | 0.82 (0.65, 1.02) | 0.08 |
|  | 24.0–27.9 (Overweight) | 2995 (34.6) | 606 (20.2) | 18.2 | 0.75 (0.69, 0.82) | < 0.001 |
|  | 28+ (Obese) | 826 (9.5) | 93 (11.3) | 10.4 | 0.43 (0.36, 0.53) | < 0.001 |
| Qi Deficiency | 18.5–23.9 (Normal) | 4503 (52) | 693 (15.4) | 16.1 | 1.0 (Reference) |  |
|  | <18.5 (Underweight) | 328 (3.8) | 72 (22.0) | 21.5 | 1.34 (1.07, 1.66) | 0.009 |
|  | 24.0–27.9 (Overweight) | 2995 (34.6) | 506 (16.9) | 18.1 | 1.13 (1.01, 1.25) | 0.03 |
|  | 28+ (Obese) | 826 (9.5) | 151 (18.3) | 19.1 | 1.19 (1.01, 1.39) | 0.03 |
| Phlegm Dampness | 18.5–23.9 (Normal) | 4503 (52) | 181 (4.0) | 4.0 | 1.0 (Reference) |  |
|  | <18.5 (Underweight) | 328 (3.8) | 2 (0.6) | 0.6 | 0.16 (0.04, 0.63) | 0.009 |
|  | 24.0–27.9 (Overweight) | 2995 (34.6) | 496 (16.6) | 15.3 | 3.84 (3.25, 4.53) | < 0.001 |
|  | 28+ (Obese) | 826 (9.5) | 276 (33.4) | 31.0 | 7.77 (6.53, 9.25) | < 0.001 |
| Yang Deficiency | 18.5–23.9 (Normal) | 4503 (52) | 543 (12.1) | 9.1 | 1.0 (Reference) |  |
|  | <18.5 (Underweight) | 328 (3.8) | 69 (21.0) | 16.9 | 1.85 (1.48, 2.32) | < 0.001 |
|  | 24.0–27.9 (Overweight) | 2995 (34.6) | 177 (5.9) | 4.7 | 0.51 (0.44, 0.60) | < 0.001 |
|  | 28+ (Obese) | 826 (9.5) | 24 (2.9) | 2.4 | 0.26 (0.17, 0.39) | < 0.001 |
| Yin Deficiency | 18.5–23.9 (Normal) | 4503 (52) | 331 (7.4) | 6.3 | 1.0 (Reference) |  |
|  | <18.5 (Underweight) | 328 (3.8) | 25 (7.6) | 6.3 | 0.99 (0.67, 1.48) | 0.98 |
|  | 24.0–27.9 (Overweight) | 2995 (34.6) | 145 (4.8) | 4.6 | 0.72 (0.60, 0.88) | < 0.001 |
|  | 28+ (Obese) | 826 (9.5) | 24 (2.9) | 2.7 | 0.43 (0.29, 0.65) | < 0.001 |
| Damp Heat | 18.5–23.9 (Normal) | 4503 (52) | 187 (4.2) | 3.9 | 1.0 (Reference) |  |
|  | <18.5 (Underweight) | 328 (3.8) | 18 (5.5) | 4.6 | 1.17 (0.74, 1.87) | 0.5 |
|  | 24.0–27.9 (Overweight) | 2995 (34.6) | 150 (5.0) | 4.5 | 1.14 (0.92, 1.41) | 0.23 |
|  | 28+ (Obese) | 826 (9.5) | 40 (4.8) | 3.9 | 0.98 (0.70, 1.37) | 0.88 |
| Qi Stagnation | 18.5–23.9 (Normal) | 4503 (52) | 134 (3.0) | 2.7 | 1.0 (Reference) |  |
|  | <18.5 (Underweight) | 328 (3.8) | 9 (2.7) | 2.0 | 0.73 (0.37, 1.44) | 0.36 |
|  | 24.0–27.9 (Overweight) | 2995 (34.6) | 55 (1.8) | 2.0 | 0.72 (0.53, 1.00) | 0.05 |
|  | 28+ (Obese) | 826 (9.5) | 18 (2.2) | 2.1 | 0.78 (0.48, 1.27) | 0.32 |
| Inherited Special | 18.5–23.9 (Normal) | 4503 (52) | 60 (1.3) | 1.2 | 1.0 (Reference) |  |
|  | <18.5 (Underweight) | 328 (3.8) | 2 (0.6) | 0.4 | 0.33 (0.08, 1.42) | 0.14 |
|  | 24.0–27.9 (Overweight) | 2995 (34.6) | 30 (1.0) | 1.1 | 0.91 (0.58, 1.42) | 0.68 |
|  | 28+ (Obese) | 826 (9.5) | 6 (0.7) | 0.7 | 0.59 (0.26, 1.38) | 0.22 |
| Blood Stasis | 18.5–23.9 (Normal) | 4503 (52) | 39 (0.9) | 0.7 | 1.0 (Reference) |  |
|  | <18.5 (Underweight) | 328 (3.8) | 5 (1.5) | 1.1 | 1.77 (0.73, 4.30) | 0.21 |
|  | 24.0–27.9 (Overweight) | 2995 (34.6) | 20 (0.7) | 0.6 | 0.86 (0.50, 1.47) | 0.58 |
|  | 28+ (Obese) | 826 (9.5) | 5 (0.6) | 0.5 | 0.78 (0.31, 1.95) | 0.59 |

1 The adjusted prevalence ratio was estimated using modified Poisson regression models with robust standard errors, adjusting for age and sex. The adjusted prevalence of body constitutions in each subgroup was calculated using marginal standardization.

**Supplementary Table S5. BMI-specific prevalence of primary body constitutions, overall and by age (N = 8,665).**

| Body constitution | Age group | Prevalence, n (%) | | | | *p* value^1^ |
| --- | --- | --- | --- | --- | --- | --- |
|  |  | **<18.5 (Underweight)** | **18.5–23.9 (Normal)** | **24.0–27.9 (Overweight)** | **28+ (Obese)** |  |
| Balanced | All | 65 (19.8) | 1163 (25.8) | 606 (20.2) | 93 (11.3) | < 0.001 |
|  | 18–29 | 10 (17.5) | 38 (14.7) | 10 (10.8) | 3 (5.3) | 0.17 |
|  | 30–49 | 15 (15.8) | 274 (21.5) | 131 (17.3) | 21 (8.7) | < 0.001 |
|  | 50–59 | 15 (26.8) | 328 (28.7) | 167 (20.1) | 25 (12.3) | < 0.001 |
|  | 60–69 | 19 (21.3) | 390 (29.2) | 212 (22.6) | 37 (16.4) | < 0.001 |
|  | 70+ | 6 (19.4) | 133 (26.8) | 86 (22.8) | 7 (7.1) | < 0.001 |
| Phlegm Dampness | All | 2 (0.6) | 181 (4.0) | 496 (16.6) | 276 (33.4) | < 0.001 |
|  | 18–29 | 0 (0.0) | 11 (4.2) | 11 (11.8) | 18 (31.6) | < 0.001 |
|  | 30–49 | 2 (2.1) | 50 (3.9) | 138 (18.2) | 86 (35.5) | < 0.001 |
|  | 50–59 | 0 (0.0) | 51 (4.5) | 141 (17.0) | 77 (37.9) | < 0.001 |
|  | 60–69 | 0 (0.0) | 49 (3.7) | 159 (17.0) | 68 (30.1) | < 0.001 |
|  | 70+ | 0 (0.0) | 20 (4.0) | 47 (12.5) | 27 (27.6) | < 0.001 |
| Yang Deficiency | All | 69 (21.0) | 543 (12.1) | 177 (5.9) | 24 (2.9) | < 0.001 |
|  | 18–29 | 4 (7.0) | 19 (7.3) | 2 (2.2) | 1 (1.8) | 0.14 |
|  | 30–49 | 17 (17.9) | 140 (11.0) | 29 (3.8) | 4 (1.7) | < 0.001 |
|  | 50–59 | 11 (19.6) | 123 (10.8) | 36 (4.3) | 9 (4.4) | < 0.001 |
|  | 60–69 | 29 (32.6) | 167 (12.5) | 76 (8.1) | 5 (2.2) | < 0.001 |
|  | 70+ | 8 (25.8) | 94 (19.0) | 34 (9.0) | 5 (5.1) | < 0.001 |
| Yin Deficiency | All | 25 (7.6) | 331 (7.4) | 145 (4.8) | 24 (2.9) | < 0.001 |
|  | 18–29 | 5 (8.8) | 19 (7.3) | 9 (9.7) | 2 (3.5) | 0.56 |
|  | 30–49 | 6 (6.3) | 105 (8.3) | 35 (4.6) | 7 (2.9) | 0.001 |
|  | 50–59 | 2 (3.6) | 86 (7.5) | 40 (4.8) | 5 (2.5) | 0.008 |
|  | 60–69 | 8 (9.0) | 90 (6.7) | 38 (4.1) | 5 (2.2) | 0.002 |
|  | 70+ | 4 (12.9) | 31 (6.2) | 23 (6.1) | 5 (5.1) | 0.46 |

1 Chi-squared tests were used to compare prevalence differences across BMI subgroups within each age group.

**Supplementary Table S6. BMI-specific prevalence of primary body constitutions, overall and by sex (N = 8,665).**

| Body constitution | Sex | Prevalence, n (%) | | | | *p* value^1^ |
| --- | --- | --- | --- | --- | --- | --- |
|  |  | **<18.5 (Underweight)** | **18.5–23.9 (Normal)** | **24.0–27.9 (Overweight)** | **28+ (Obese)** |  |
| Balanced | All | 65 (19.8) | 1163 (25.8) | 606 (20.2) | 93 (11.3) | < 0.001 |
|  | Male | 22 (21.2) | 456 (30.0) | 298 (21.7) | 40 (10.5) | < 0.001 |
|  | Female | 43 (19.2) | 707 (23.7) | 308 (19.0) | 53 (11.9) | < 0.001 |
| Phlegm Dampness | All | 2 (0.6) | 181 (4.0) | 496 (16.6) | 276 (33.4) | < 0.001 |
|  | Male | 0 (0.0) | 99 (6.5) | 287 (20.9) | 154 (40.5) | < 0.001 |
|  | Female | 2 (0.9) | 82 (2.7) | 209 (12.9) | 122 (27.4) | < 0.001 |
| Yang Deficiency | All | 69 (21.0) | 543 (12.1) | 177 (5.9) | 24 (2.9) | < 0.001 |
|  | Male | 18 (17.3) | 104 (6.8) | 41 (3.0) | 5 (1.3) | < 0.001 |
|  | Female | 51 (22.8) | 439 (14.7) | 136 (8.4) | 19 (4.3) | < 0.001 |
| Yin Deficiency | All | 25 (7.6) | 331 (7.4) | 145 (4.8) | 24 (2.9) | < 0.001 |
|  | Male | 11 (10.6) | 63 (4.1) | 34 (2.5) | 4 (1.1) | < 0.001 |
|  | Female | 14 (6.2) | 268 (9.0) | 111 (6.9) | 20 (4.5) | 0.002 |

1 Chi-squared tests were used to compare prevalence differences across BMI subgroups within each age group.
